# Supplementary material for: Single-Cell Perturbations Reveal Selective Modulation of Causal Connectivity During Decision-Making
Source: bioRxiv. 2026 Apr 8:2026.04.07.716761. Preprint. [Version 1] doi: 10.64898/2026.04.07.716761 (PMC13081855; doi:10.64898/2026.04.07.716761)
Supplement: Supplement 1 [file NIHPP2026.04.07.716761v1-supplement-1.pdf]

bioRxiv preprint doi: https://doi.org/10.64898/2026.04.07.716761; this version posted April 8, 2026. The copyright holder for this preprint (which was not certified by peer review) is the author/funder, who has granted bioRxiv a license to display the preprint in perpetuity. It is made available under aCC-BY 4.0 International license.

SUPPLEMENTAL INFORMATION

bioRxiv preprint doi: https://doi.org/10.64898/2026.04.07.716761; this version posted April 8, 2026. The copyright holder for this preprint (which was not certified by peer review) is the author/funder, who has granted bioRxiv a license to display the preprint in perpetuity. It is made available under aCC-BY 4.0 International license.

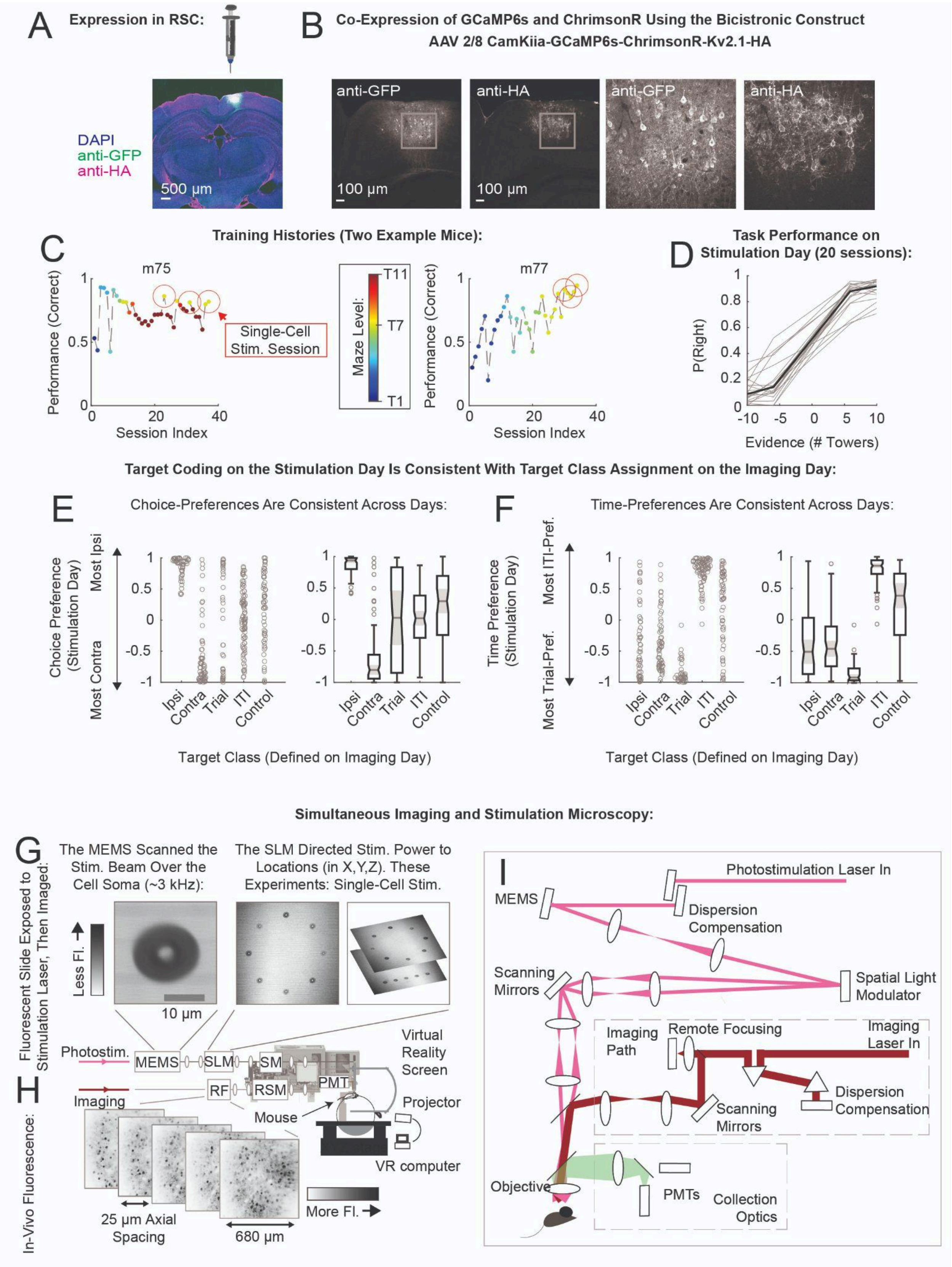

bioRxiv preprint doi: https://doi.org/10.64898/2026.04.07.716761; this version posted April 8, 2026. The copyright holder for this preprint (which was not certified by peer review) is the author/funder, who has granted bioRxiv a license to display the preprint in perpetuity. It is made available under aCC-BY 4.0 International license.

Figure S1. Technical Details, Related to Figure 1.

A,B. We used a bicistronic construct to co-express the calcium indicator GCaMP6s and the soma-localized opsin ChrimsonR-Kv2.1 using the CamKiiα promoter[1–5].
A. Image of viral expression targeting RSC.
B. Higher magnification of the image in panel A; anti-GFP and anti-HA expression shown separately. Right panels: Expanded view of the boxed region in left panels. Neurons co-express opsin and indicator; ChrimsonR-Kv2.1 expression (anti-HA) appears less prominent in the background.
C. Performance correct (on all trials per session, including warm up and aberrant trials) vs. session index, for two representative mice. Single-cell stimulation sessions are indicated with red circles. Color indicates maze difficulty. All mice used in the single-cell stimulation experiments were able to perform the no-distractors version (T7) of the ATT well (see panel D). Mice were trained on the full ATT as far in maze difficulty as they could progress (Methods : Behavioral Training). Training histories for all nine mice are shown in Fig. S14.
D. Performance per session, in trials passing behavioral criteria, but not selected for attentive state (see Fig. S4 for attentive state selection; individual 20 sessions in 9 mice in gray, median in black). Correct performance: median, 88%; range, 72–96%.
E,F. Choice (**E**) and time (**F**) preferences of neurons close to the stimulation point (directly activated by stimulation), evaluated on nonstimulation trials on the stimulation day (Methods : Choice- and Time-Preferences of Neurons), organized by the target class definition on the imaging day.
Left panel: swarm chart;
Right panel: statistics (Matlab's **boxchart**: shaded region indicates 95% C.I. on median; box indicates lower and upper quartiles).
For a more detailed analysis of the encoding properties of target classes using generalized linear models, see Fig. S2.
G–I. Simultaneous imaging and stimulation microscope.
G. Stimulation path deflections were calibrated by bleaching a fluorescent slide, which was then imaged by the imaging path (top panels). The photostimulation path used a MEMS scanner to create a ~3kHz spiral scan over a ~17 µm wide annulus (top left), which was directed towards a target neuron with the spatial light modulator (SLM; top middle and top right). Scanning mirrors (SM) were centered in these experiments.
H. Averaged fluorescence (mean image from Suite2p[6]) from a recorded session in-vivo (bottom panels, inverted grayscale indicates brighter cells as darker). We recorded from five planes with 25 µm axial spacing in these experiments. The imaging path incorporated a remote focusing (RF) unit consisting of a paired secondary objective and voice coil[7] to provide high quality optical performance over a range of axial deflections. Resonant scanning mirrors (RSM) and photomultiplier tubes (PMT) completed the imaging path.
I. Schematic of the optical pathways, described in greater detail in Methods.

bioRxiv preprint doi: https://doi.org/10.64898/2026.04.07.716761; this version posted April 8, 2026. The copyright holder for this preprint (which was not certified by peer review) is the author/funder, who has granted bioRxiv a license to display the preprint in perpetuity. It is made available under aCC-BY 4.0 International license.

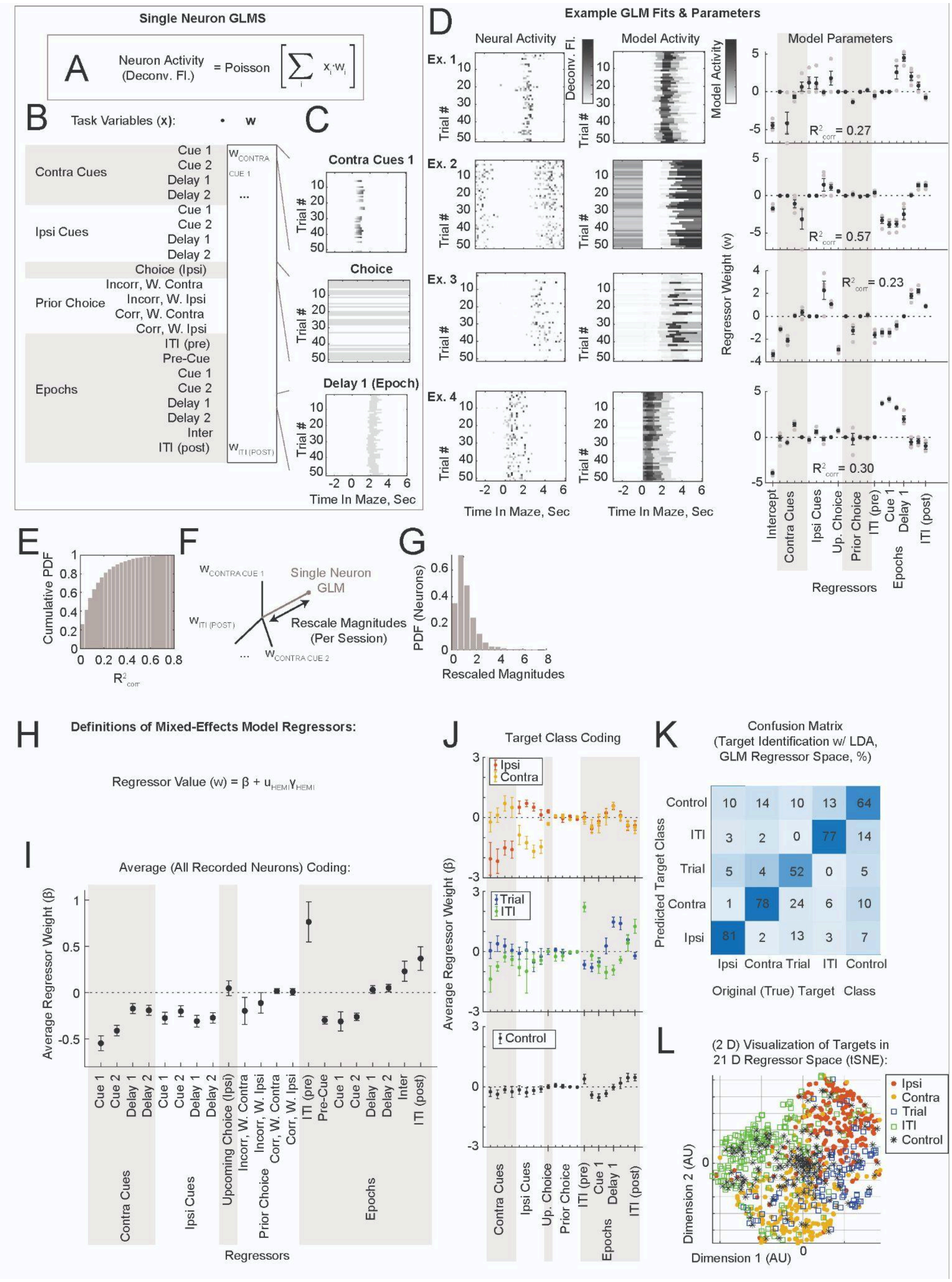

bioRxiv preprint doi: https://doi.org/10.64898/2026.04.07.716761; this version posted April 8, 2026. The copyright holder for this preprint (which was not certified by peer review) is the author/funder, who has granted bioRxiv a license to display the preprint in perpetuity. It is made available under aCC-BY 4.0 International license.

Figure S2. Encoding Models for Single Neurons, Related to Figure 1.

We regressed the activity of a single neuron against task variables with generalized linear models (Methods: Single Neuron Encoding Models).

A. Formula describing the encoding model. Individual GLMs were fit to the activity (deconvolved fluorescence estimated using the OASIS algorithm[8]) of a single neuron on non-stimulation trials.

B. The task variables used as regressors in the model.

C. Examples of regression inputs (heat maps of input values, trial (row) by time (column)). All task variables were normalized across trials to a range of 1.

Top panel: Sensory evidence input varied within-epoch and across-trials. Sensory evidence was included in the model in 8 terms (top 8 rows in panel B), corresponding to which side the cues were presented on and conditioned to the four maze-traversing epochs (Early Cue, Late Cue, Early Delay, Late Delay). These values were set to 0 in other epochs, and scaled to the 0–1 range within-epoch.

Middle panel: Choice on the current trial and the four prior choice conditions (binary values for correct/incorrect and went-ipsi/went-contra) was represented with constant terms per trial.

Bottom panel: The ongoing epoch was represented with a binary indicator (other epoch, 0; this epoch, 1).

D. Four example neurons (rows Ex. 1: Ex. 4). These were randomly selected from all neurons with $R^2 >$ 0.08.

Left column: Heat maps of the deconvolved neural activity.

Center column: Predicted Model Activity.

Right column: Estimates of the regressor coefficients (w) per neuron. Gray: values from individual 4-fold estimates, black and error bars are mean and standard error across 4-folds. The intercept was not used in subsequent analyses.

E. The distribution of variance explained across all model fits with sufficient samples and positive variance explained (4862 out of a total of 6695 neurons, 20 sessions).

F. In order to compare regressors across sessions, we rescaled all regressor vectors so that the median magnitude within-session would be unity.

G. Distribution of rescaled magnitudes across sessions.

H. Definitions of regressors (related to panels I,J). The fixed-effects coefficients (□) provide estimates of the average values of the encoding-model regressors w, allowing for variability across hemispheres.

I. Average encoding of regressors across all neurons (4862 neurons, 11 hemispheres). Black dots and error bars indicate the estimate and 95% C.I.

J. Average encoding of regressors within target class neurons recruited for stimulation, organized by target class (data from imaging day recording).

Top panel: Ipsi (red) and Contra (gold) target classes.

Middle panel: Trial (blue) and ITI (green) target classes.

Bottom panel: Control target class. The coding of Control neurons seems fairly similar to the average coding profile in panel I.

K,L. We used the encoding models of each target, and the assigned target class, to assess the separability of target classes by their encoding.

K. Classifier performance, shown as a confusion matrix, averaged over 1000x 2-fold cross-validated fits. Linear classifiers (Matlab's **fitcdiscr**) were trained to separate target classes in the regressor space of the encoding models, and tested on a withheld half of targets. The test targets which did not have a classification probability >50% for any target class (16% of all targets) were excluded from this analysis.

L. Two-dimensional embedding of the 21-dimensional target coding profiles (tSNE, Euclidean distance). This embedding was agnostic to the target class label.

The classes with opposing preferences separated well from each other (panel K; Ipsi and Contra; Trial and ITI).

bioRxiv preprint doi: https://doi.org/10.64898/2026.04.07.716761; this version posted April 8, 2026. The copyright holder for this preprint (which was not certified by peer review) is the author/funder, who has granted bioRxiv a license to display the preprint in perpetuity. It is made available under aCC-BY 4.0 International license.

Trial targets were frequently misidentified as Ipsi or Contra targets (panel K). Trial targets tend to have choice-preferences (Fig. S1E), and time-preferences of Trial and Ipsi / Contra neurons behave similarly (panel J; Trial targets tend to peak a little later than Ipsi / Contra targets).

Control targets were identified as Control in ~65% of cases (panel K). Most of these are a core non-coding group of neurons (dense core of black asterisks in the center of panel L). The remaining ~35% fall into the remaining categories, suggesting that the excitatory layer 2/3 RSC population could be categorized as ~10% Ipsi, ~10% Contra, ~5% Trial, ~15% ITI.

For these reasons, throughout this paper we focused on three target class-based comparisons: Ipsi to Contra, Trial to ITI, and all four task-encoding target classes to Control.

bioRxiv preprint doi: https://doi.org/10.64898/2026.04.07.716761; this version posted April 8, 2026. The copyright holder for this preprint (which was not certified by peer review) is the author/funder, who has granted bioRxiv a license to display the preprint in perpetuity. It is made available under aCC-BY 4.0 International license.

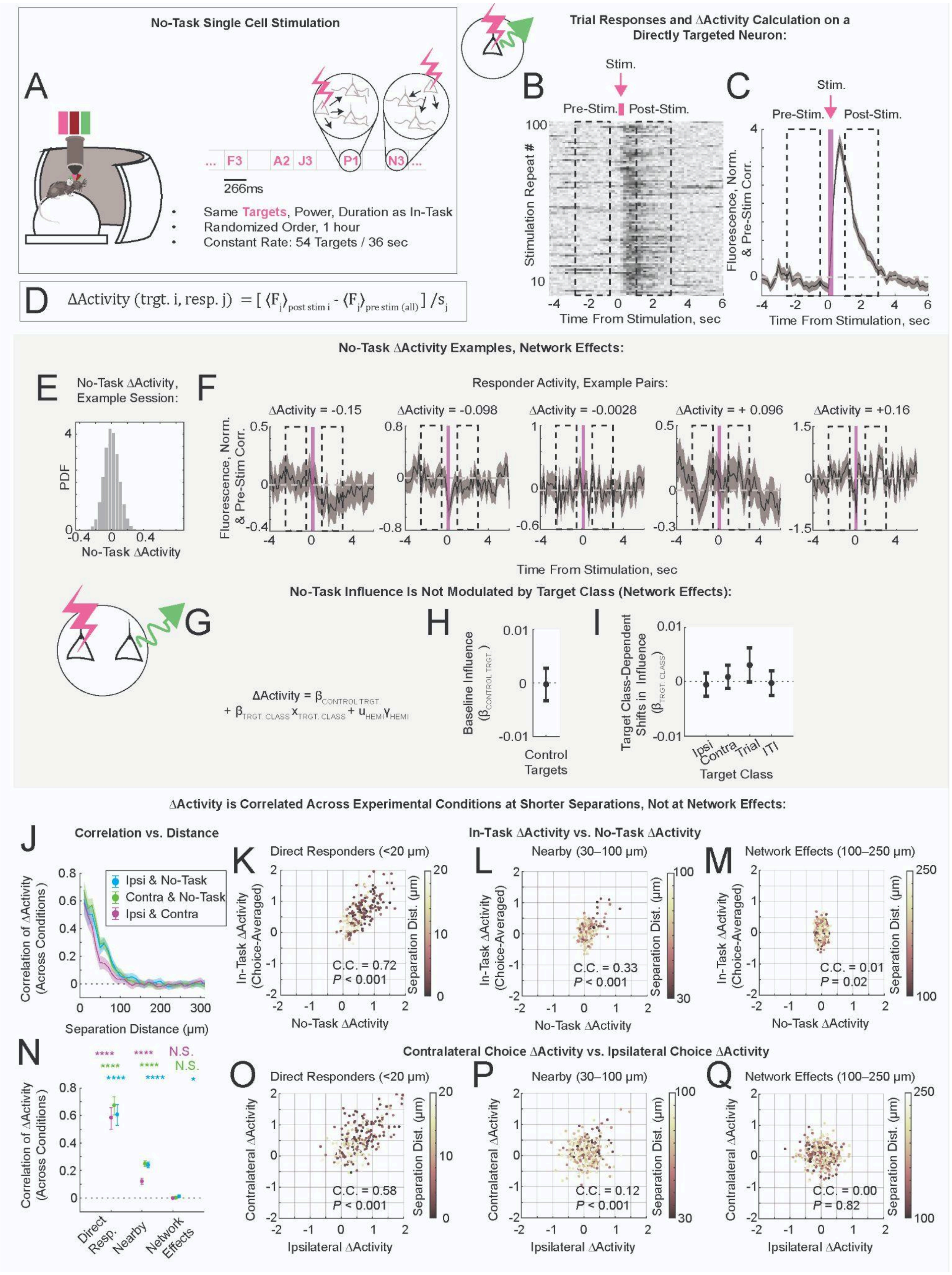

bioRxiv preprint doi: https://doi.org/10.64898/2026.04.07.716761; this version posted April 8, 2026. The copyright holder for this preprint (which was not certified by peer review) is the author/funder, who has granted bioRxiv a license to display the preprint in perpetuity. It is made available under aCC-BY 4.0 International license.

Figure S3. Measurement and Characterizations of No-Task Causal Connectivity, Related to Figures 1–4.

In-task measurements of causal connectivity (Fig. 2) were followed with a no-task stimulation protocol in a subset of sessions (11 out of 20).

A. Following the in-task stimulation session, mice remained head-fixed on the spherical treadmill, while the VR screen was dark. Optical stimulation parameters were identical to those used in the in-task stimulation protocol (target location, duration of exposure, power). Stimulation order was randomized in the following way: every 36 seconds, each target was stimulated once. The order of stimulation within each 36 second block was randomized within each block (Methods : No-Task Stimulation).

B,C. As in Fig. 2, calculation of ΔActivity is illustrated using a directly stimulated neuron for clarity.

B. Fluorescence of a responding neuron (heat map, inverted grayscale colorbar such that darker = more activity) in response to 100 stimulation repeats. The effects of brief 266ms long stimulation (magenta bar) were evaluated comparing the averaged activity in post-stimulation (1–3 seconds following stim, dashed box) and pre-stimulation (-2.5 to -0.5 seconds preceding stim, dashed box) periods. Note that the pre-stimulation periods were not target-specific and included all pre-stimulation time bins in the session.

C. Averaged fluorescence in response to stimulation, with pre-stimulation, stimulation and post-stimulation periods indicated. Shaded area indicates STE across 100 stimulation repeats.

D. Definition of no-task ΔActivity. Stimulations of targets closer than 25 μm to the responder were excluded from the calculation of the normalization coefficient. Since most stimulation targets are >> 25 μm away, the normalization coefficient was estimated over ~5000 numbers per target-responder pair (54 targets, 100 stimulations each per session).

E. No-task distribution of ΔActivity (network effects range).

F. Examples of ΔActivity (shown: stimulation-averaged responder activity vs. time following stimulation).

G–I. To check if there were target class-dependent shifts in influence (similar to the epoch-conditioned, target class-dependent shifts in Fig. 5E), we used a mixed-effects model (**G**) to estimate the dependence of influence on target class, using ΔActivity measured in the no-task condition. Neither the baseline influence (Control target class stimulation, **H**), nor the target class-dependent shifts in influence (**I**) significantly deviated from zero.

J–O. Correlation coefficients of ΔActivity across experimental conditions. These are measures of: how consistent is the causal connectivity when measured across conditions? Perhaps surprisingly, ΔActivity measured during ipsilateral choices was less correlated with ΔActivity measured during contralateral choices, than with ΔActivity measured in the no-task condition.

J. Correlation of ΔActivity vs. separation distance (20 μm bins).

K–M. Choice-averaged in-task ΔActivity vs. no-task ΔActivity (individual pair = point), grouped by distance (**K**, <20 μm; **L**, 30–100 μm; **M**, 100–250 μm). For target-responder pairs in the nearby and network effects separation ranges, we subselected a random 500 pairs to plot. Correlation coefficient and corresponding significance value for all pairs indicated (# of pairs in panel K, 271; L, 8 555; M, 53 662 pairs).

N. Correlation of ΔActivity vs. separation distance (same as panel J), grouped into the separation categories Direct Resp., Nearby, and Network Effects.

O–Q. Comparison of ipsilateral to contralateral in-task ΔActivity. Same pairs, same organization as in panels K–M.

Significance values in panels K–Q were multiple comparisons corrected (Bonferroni, 6X).

bioRxiv preprint doi: https://doi.org/10.64898/2026.04.07.716761; this version posted April 8, 2026. The copyright holder for this preprint (which was not certified by peer review) is the author/funder, who has granted bioRxiv a license to display the preprint in perpetuity. It is made available under aCC-BY 4.0 International license.

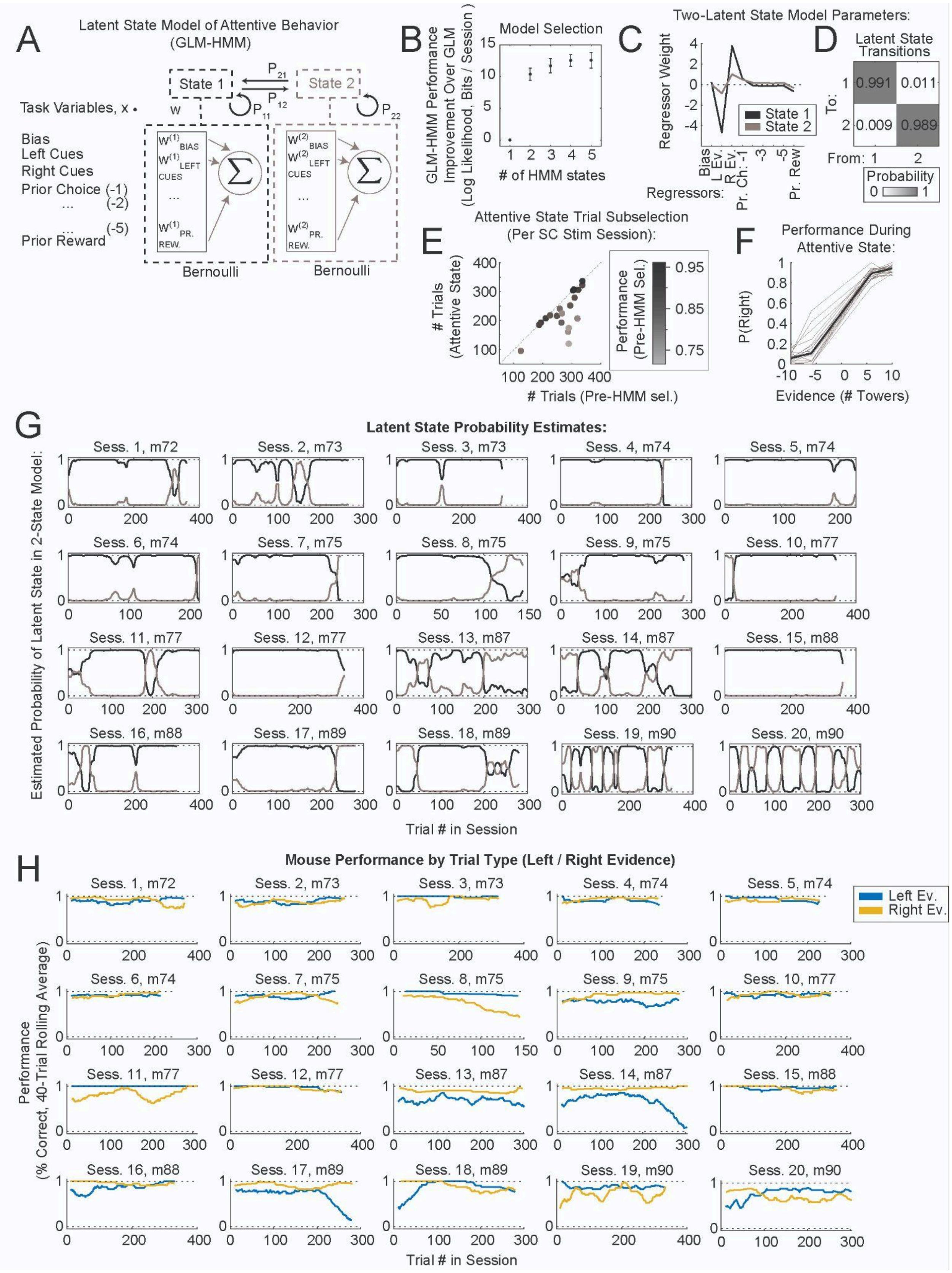

bioRxiv preprint doi: https://doi.org/10.64898/2026.04.07.716761; this version posted April 8, 2026. The copyright holder for this preprint (which was not certified by peer review) is the author/funder, who has granted bioRxiv a license to display the preprint in perpetuity. It is made available under aCC-BY 4.0 International license.

Figure S4. Attentive State Characterization Using a Two-Latent State GLM-HMM Model of Behavior, Related to Figures 3–5.

A. Schematic of the latent state model of behavior implemented (see also Ref. [9]). This model aimed to capture the dependence of animal choice on task variables (sensory evidence, prior choice) using multiple latent states (in this example, the regressor weights w within the black and gray squares have superscripts indicating the state), and a markov transition matrix between them. The model fitting procedure is described in Methods : Latent State Model of Task Engagement, and closely followed Ref. [9].
B. Cross-validated performance of models vs. number of latent states.
C. Regressor weights per state, for the two-latent state model. Prior choice (went-right / went-left) had five regressors corresponding to the number of trials back (i.e. -1 is the trial immediately preceding the current one). The two-latent state model is simple to interpret: state 1 is more attentive, state 2 is less attentive with more weight in the prior choice terms.
D. Probabilities of transitions between the latent states, as estimated by the model. Low probabilities of a transition between different states reflect the slower time scales captured by the model.
E. # of trials classified as attentive vs. total # of trials (individual points are sessions), colored by performance estimated pre-selection.
F. Performance in the attentive state on stimulation sessions. Gray lines are individual sessions, black is median over sessions.
G. Model-estimated probabilities of latent states, per session, shown for the twenty single-cell stimulation sessions analyzed in this paper.
H. Mouse performance on all trials (prior to attentive state selection, averaged in a 40-trial rolling bin), per trial type, is shown for comparison to panel D.

bioRxiv preprint doi: https://doi.org/10.64898/2026.04.07.716761; this version posted April 8, 2026. The copyright holder for this preprint (which was not certified by peer review) is the author/funder, who has granted bioRxiv a license to display the preprint in perpetuity. It is made available under aCC-BY 4.0 International license.

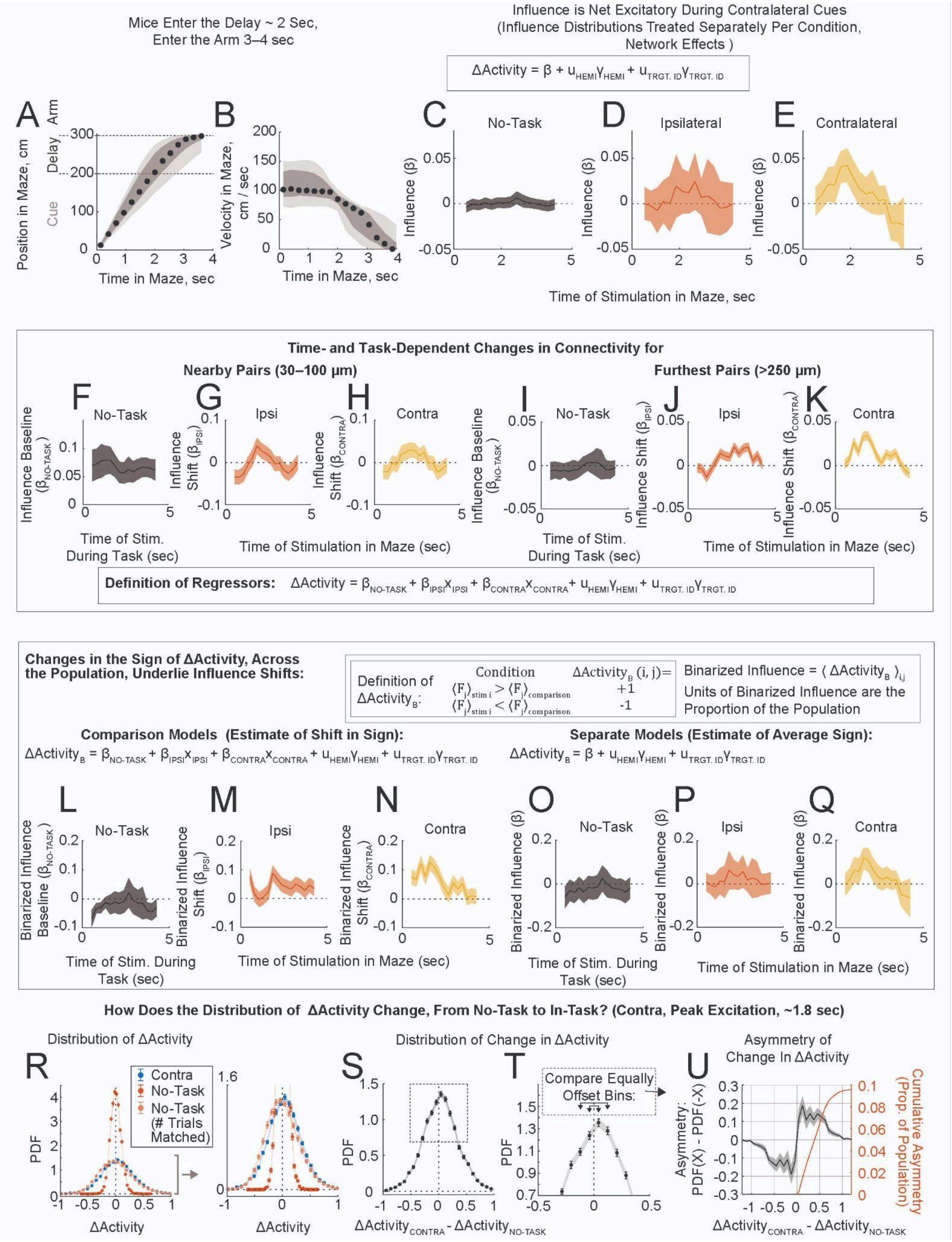

bioRxiv preprint doi: https://doi.org/10.64898/2026.04.07.716761; this version posted April 8, 2026. The copyright holder for this preprint (which was not certified by peer review) is the author/funder, who has granted bioRxiv a license to display the preprint in perpetuity. It is made available under aCC-BY 4.0 International license.

Figure S5. Supplemental Information Related to Figure 3.

A,B. Median position (**A**) and velocity (along the stem of the maze, **B**), across sessions, in the maze. Position and velocity were evaluated at the stimulation onset times of targets (median across sessions, black dot, 2nd and 3rd quartiles dark shaded area, 95% range, light shaded area). Entry into the Delay was typically around 2 seconds from the start of the maze, the time point at which animals also started to slow down to navigate the upcoming turn.
C–E. Influence (□), vs. time of stimulation in the maze, estimated using target-responder pairs in the network effects range (Model 1.3, Table 1). These regressors were estimated separately per experimental condition; data and conventions (shaded areas indicate 95% C.I.) are the same as in Fig. 3I–K. There were no significant trends in the no-task or ipsilateral influence. Peak deviation in contralateral influence (~1.8 seconds in panel E) was □ = 0.042, ****P_{CORR} = 6.1e-05 (Bonferroni 9X).
F–H. Influence baseline (**F**) and task-dependent shift (**G,H**) vs. time of stimulation in the maze, analogous to the main text (Fig. 3I–K), for target-responder pairs nearby (30–100 µm, 8 hemispheres, 378 targets, 8 555 pairs).
It is unclear from these panels: is this a noisy version of the same trends we observed in the network effects range? Or is this a common, choice-invariant trend? Because our sampling was greatly reduced at closer separations (and for the other reasons mentioned in the main text), we focused on the target-responder pairs at network effects separations throughout this paper.
I–K. Influence baseline (**I**) and task-dependent shift (**J,K**) vs. time of stimulation in the maze, analogous to the main text (Fig. 3I–K), for target-responder pairs at greater separation distances (>250 µm, 8 hemispheres, 389 targets, 79 723 pairs).
The trends in panels I–K look qualitatively similar to those reported in the main text, for network effects separations.
L–Q. Analyses of the binarized ΔActivity and binarized influence (definitions in inset). We binarized ΔActivity to a +1/-1 notation ($\Delta\text{Activity}_{\mathbf{B}}$). This quantity is advantageous because the units of the binarized influence are the proportion of the population and hence easy to interpret. The disadvantage is that the normalization of ΔActivity has been removed, and so noisy pairs contribute to estimates of the binarized influence on the same scale as less noisy pairs.
L–N. Analyses of the binarized influence (network effects range; Model 1.4, Table 1, compare to Fig. 3I–K).
L. Binarized influence baseline (estimated in the no-task condition) vs. time of stimulation in the maze.
M. Shifts in binarized influence vs. time of stimulation in the maze, ipsilateral choice. At ~1 sec, there is no significant change in the proportion of signed interactions (□ = 0.00, *n.s.*). At ~1.8 sec, ~9% of the proportion of pairs shifts excitatory (□ = 0.09, ****P_{CORR} = 1.8e-10, Bonferroni 9X).
N. Shifts in binarized influence vs. time of stimulation in the maze, contralateral choice. Both ~1 and ~1.8 sec have >10% shifts in the proportion of pairs that are excitatory (1 sec: □ = 0.12, ****P_{CORR} = 6.3e-19; 1.8 sec: □ = 0.11, ****P_{CORR} = 4.5e-15, Bonferroni 9X).
O–Q. Binarized influence vs. time of stimulation in the maze; regressors estimated separately for the no-task (**O**), ipsilateral (**P**) and contralateral (**Q**) conditions. Data and approach (estimating regressors per condition) is the same as in panels C–E (Model 1.5, Table 1).
The peak excitation during contralateral cue presentation (at ~1.8 seconds, panel Q) corresponded to ~11% more excitatory than inhibitory pairs in the population (□ = 0.11, ***P_{CORR} = 5.4e-04, Bonferroni 9X).
R–U. Changes in the distribution of ΔActivity, shown for peak contralateral offsets (~1.8 seconds, stimulation bins 6 through 9, network effects, 11 200 pairs).
R. Distributions of ΔActivity for in-task contralateral (blue), and no-task (red). The right panel provides an expanded view. Error bars are standard deviations over resampled pairs (with replacement). No-task ΔActivity was estimated by comparing 100 post-stimulation responses to 3–5 x 10^3 pre-stimulation responses. In-task ΔActivity compared ~20 stim to ~20 comparison trials. We suspected that differences

bioRxiv preprint doi: https://doi.org/10.64898/2026.04.07.716761; this version posted April 8, 2026. The copyright holder for this preprint (which was not certified by peer review) is the author/funder, who has granted bioRxiv a license to display the preprint in perpetuity. It is made available under aCC-BY 4.0 International license.

in sampling between no-task and in-task conditions were the leading cause of the different widths in the distributions of ΔActivity (compare red to blue). To check for this, we resampled no-task ΔActivity, downsampled to match the number of samples in the in-task measurement (N_{STIM}, $N_{COMPARISON}$). Specifically, we selected $(1/N_{STIM} + 1/N_{COMPARISON})^{-1}$ of the available no-task post-stimulation responses (numbers matched per target-responder pair), and did not downsample the pre-stimulation responses (light red). Error bars for the downsampled estimates were standard deviations over 100X different post-stimulation resamples. Notice that this corresponds to a ~10X downsampling of the no-task stimulation data. Following this correction for sampling, the widths of the distributions of ΔActivity became comparable between contra and no-task conditions (compare light red to blue), while the means remained offset.

S. Distribution of the change in ΔActivity (contra - no-task), per pair.

T. Expanded view of the distribution in panel S.

U. Asymmetry in PDF (left y-axis) shows increased weight in positive values. The cumulative asymmetry (right y-axis) shows the excess proportion of the population (reaching 9.8%, right axis). This is similar to the coefficient of ~11% estimated in panels N,Q.

bioRxiv preprint doi: https://doi.org/10.64898/2026.04.07.716761; this version posted April 8, 2026. The copyright holder for this preprint (which was not certified by peer review) is the author/funder, who has granted bioRxiv a license to display the preprint in perpetuity. It is made available under aCC-BY 4.0 International license.

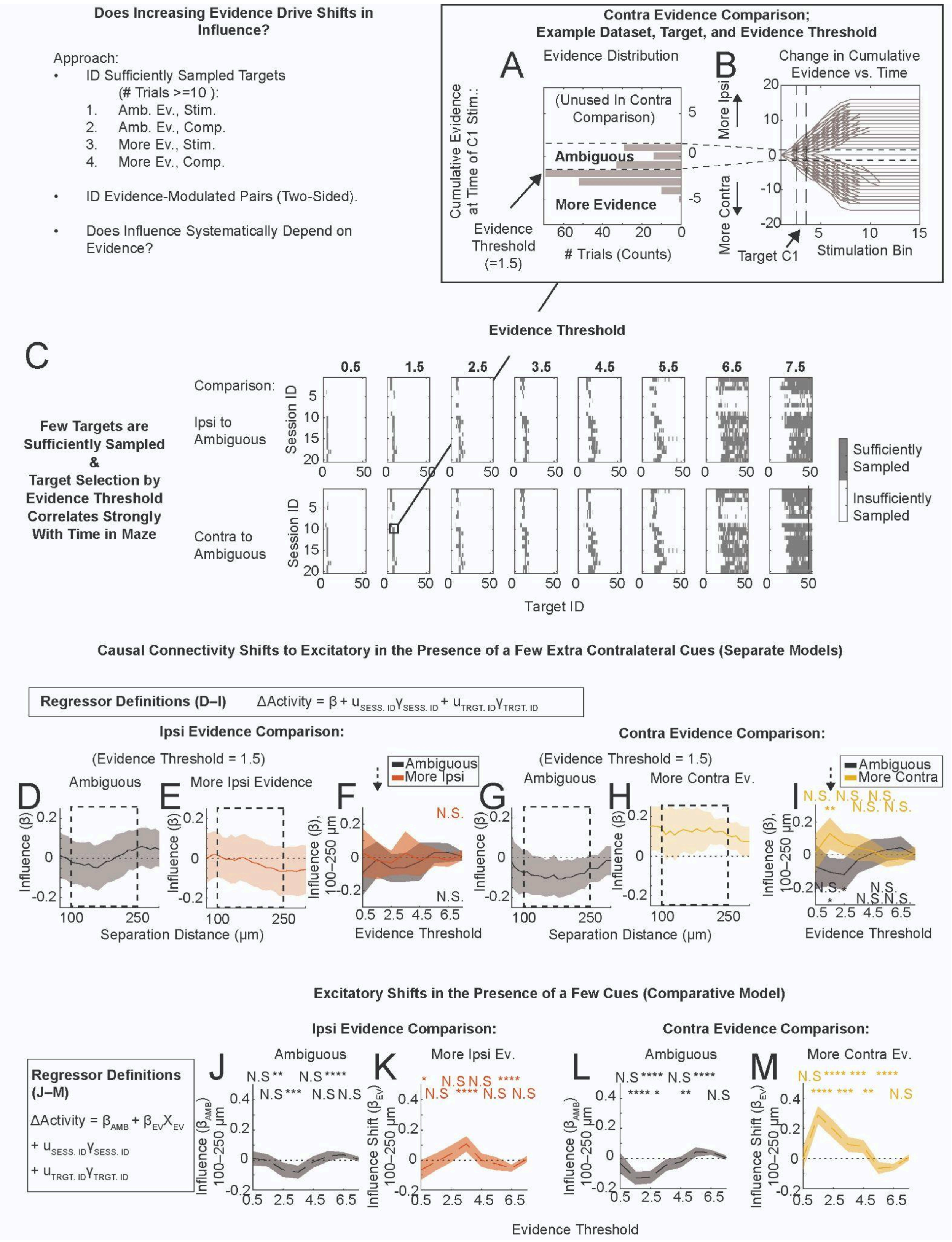

Figure S6. Contralateral Cues Drive Net Excitation, Related to Figure 4.

bioRxiv preprint doi: https://doi.org/10.64898/2026.04.07.716761; this version posted April 8, 2026. The copyright holder for this preprint (which was not certified by peer review) is the author/funder, who has granted bioRxiv a license to display the preprint in perpetuity. It is made available under aCC-BY 4.0 International license.

We probed whether influence depends on evidence. This analysis was constrained to comparing ΔActivity across evidence conditions within target-responder pairs. This constraint constrains parameters related to navigation, such as the timing of stimulation relative to maze traversal. Because we were very limited in sampling with this analysis, we included all trials that passed the behavioral trial selection criteria (and did not condition trials by attentive state or correct outcome, as in all other analyses in this paper).

A,B. We first identified the targets that were sufficiently sampled for comparison. At an evidence threshold of 1.5, target C1 in the 12th session was sufficiently sampled in both ambiguous and contralateral more-evidence conditions.

A. Histogram of the instantaneous evidence at the time of C1 stimulation. Evidence threshold indicated with dashed lines. Trials with a cumulative two or more contralateral cues at the time of stimulation were classified into the “more evidence” trials; trials with a cumulative 0 or 1 cue (on either side) were classified as “ambiguous” trials. Trials with 2 or more ipsilateral cues were not used in this (contra) comparison.

B. Instantaneous evidence vs. stimulation time bin for all trials in this session; stimulation bin of target C1 and evidence threshold are indicated with dashed lines.

C. Targets that were sufficiently sampled per evidence threshold, shown per session, across comparisons.

D–M. From the adequately sampled targets, we then identified target-responder pairs with significant evidence-dependent modulation of ΔActivity (by comparing to a resampled null distribution, Methods : Analysis of Evidence-Dependent Shifts in Causal Connectivity). The analysis of influence estimated using significantly modulated pairs is shown in panels D–M.

D–I. Data was matched within comparisons (i.e. panels D and E are calculated using the same target-responder pairs), regression models were fit separately per condition (Model 2.0, Table 1).

D,E. Influence vs. distance, 100 μm rolling bin, for ambiguous (**D**) and more ipsilateral evidence (**E**) conditions, evidence threshold = 1.5; 17 sessions, 47 targets, 789 significantly modulated pairs.

F. Influence (network effects) vs. evidence threshold, ipsilateral comparisons.

G,H. Influence vs. distance, for ambiguous (**G**) and more contralateral evidence (**H**) conditions, evidence threshold = 1.5; 18 sessions, 52 targets, 697 pairs.

I. Influence (network effects) vs. evidence threshold, contralateral comparisons.

J–M. Estimates of evidence-dependent shifts in influence (network effects) vs. evidence threshold. Same data as in panels D–I, regression models (inset) were fit to both conditions to capture evidence-dependent offsets (Model 2.1, Table 1).

J,K. Influence baseline (β_{AMB}, **J**) and evidence-dependent shift (β_{EV}, **K**) in the ipsi evidence comparison, vs. evidence threshold.

L,M. Influence baseline (β_{AMB}, **L**) and evidence-dependent shift (β_{EV}, **M**) in the contra evidence comparison, vs. evidence threshold.

bioRxiv preprint doi: https://doi.org/10.64898/2026.04.07.716761; this version posted April 8, 2026. The copyright holder for this preprint (which was not certified by peer review) is the author/funder, who has granted bioRxiv a license to display the preprint in perpetuity. It is made available under aCC-BY 4.0 International license.

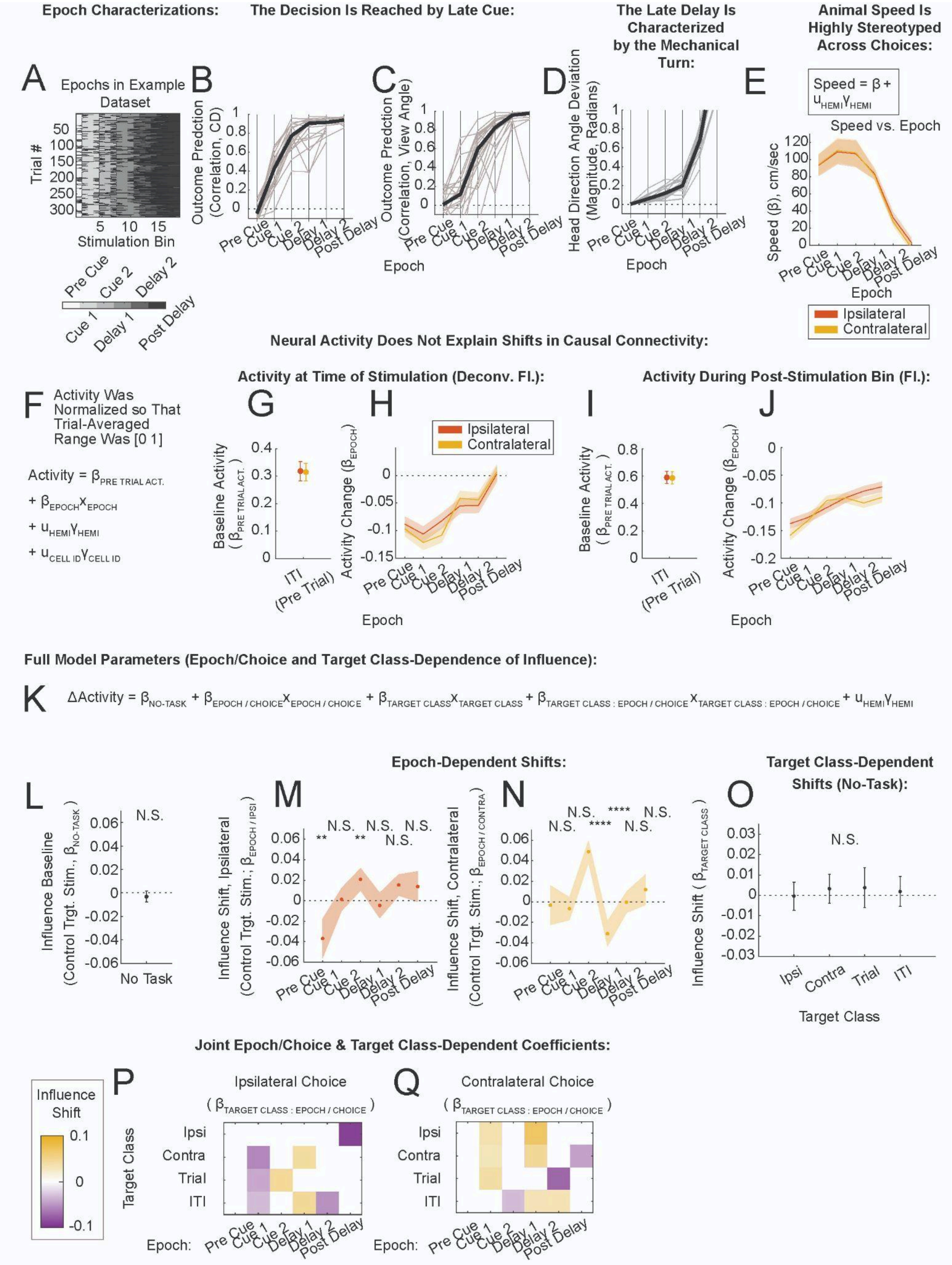

bioRxiv preprint doi: https://doi.org/10.64898/2026.04.07.716761; this version posted April 8, 2026. The copyright holder for this preprint (which was not certified by peer review) is the author/funder, who has granted bioRxiv a license to display the preprint in perpetuity. It is made available under aCC-BY 4.0 International license.

Figure S7. Epoch Characterizations and the Full Task-Dependent Model of Influence, Related to Figure 4.

A. Epoch definitions, evaluated per stimulation bin per trial (shown is an example session). The Cue period was identified on each trial, using the first and last stimulation bins within which the number of cues on the right or left hand side was changing. This time period was split in half to define the Early Cue and Late Cue epochs. The Delay period was similarly identified immediately following the Cue period until arm entry, and similarly split in half by time to define the Early and Late Delay epochs.
B. Correlation of the choice-coding projection with outcome, per epoch (gray lines are individual sessions, black is median). To calculate this, we randomly selected half of the correct went-right and correct went-left trials to calculate the z-scored choice preference per neuron per time bin (a matrix N_{CELLS} X N_{TIME} in size). We then projected the withheld-trial fluorescence onto the z-scored choice preference to estimate the choice-coding projection, per trial per time bin. To convert time-dependence to epoch-dependence, we averaged the projection over time bins belonging to the same epoch (within each trial), to obtain the choice-coding projection, per trial per epoch. This was then correlated with outcome.
C. Correlation of head view angle with choice across epochs. In this case we averaged head direction angle within epoch first, to obtain head direction angle per epoch per trial. Similarly to panel B, we used a randomly selected half of the correct went-right and correct went-left trials to calculate the average trend in head direction angle vs. epoch; projected the withheld-trial head direction angle onto this trend, and correlated the projection with outcome.
D. Absolute value of the head direction angle (radians) vs. epoch.
E. Velocity (down the stem of the T) across animals, ipsilateral and contralateral choices in red and gold, respectively, estimated as the fixed-effects coefficient in a mixed-effects model (inset). Shaded areas are 95% C.I. Running speeds were indistinguishable across choices in early epochs, until the Post-Delay epoch (e.g. two sample t-test, ipsi vs. contra, Early Cue, n.s.; Post-Delay, $P = 0.02$. Full statistics in Table S1). This comparison indicates that running speed could not explain choice-dependent differences in causal connectivity (which were found primarily during the Early Cue). The time courses of running speed and task-dependent shifts in connectivity also did not correlate cleanly: Running speed was consistently high from Pre Cue through Late Cue; while shifts in connectivity started during Cue onset, peaked in the Late Cue and were significant in the Early Delay.
F–J. Trends in neural activity vs. epoch, evaluated on attentive, nonstimulation trials.
F. Definitions of regressors in the epoch-dependent models of neural activity. Activity within-epoch was treated as the sum of a baseline term (measured within the ITI preceding trial start), and an epoch-dependent shift. Prior to this analysis, neural activity was normalized within each neuron across trials so that the trial-averaged range across time bins would be in the 0 to 1 range per neuron. As elsewhere, shaded areas are 95% C.I.
G,H. Neural activity baseline (**G)** and epoch-dependent shifts (**H**). We used deconvolved fluorescence here to improve the time resolution of the estimate.
I,J. Neural activity baseline (**I)** and epoch-dependent shifts (**J**), evaluated in the post-stimulation time bin (the same time bin used to calculate influence, i.e. 1–3 seconds following the epoch). Here we used the raw fluorescence for consistency with the calculation of influence.
In all relevant trends of neural activity (panels G–J), neural activity was minimized in the Pre Cue / Early Cue, and rose gradually as the animals progressed through the maze. These results are similar to those obtained from population averages over the time-dependent encoding terms in single neuron GLMs (Fig. S2I). These trends in population-level activity do not clearly correlate with task- or choice- dependent changes in connectivity (Figs. 3–5). Speculatively, epoch-dependent changes in the global activity could play a role in setting the operating points of inhibitory interneuron circuitry, which could lead to changes in causal connectivity (see Fig. 6, Discussion).

bioRxiv preprint doi: https://doi.org/10.64898/2026.04.07.716761; this version posted April 8, 2026. The copyright holder for this preprint (which was not certified by peer review) is the author/funder, who has granted bioRxiv a license to display the preprint in perpetuity. It is made available under aCC-BY 4.0 International license.

K–Q. The features and estimates of a comprehensive mixed-effects model of influence measured in both no-task and in-task conditions (Model 3.1, Table 1). Dots indicate estimates; asterisks indicate significance values following multiple comparisons corrections (30X, Bonferroni); and shaded areas indicate uncorrected 95% C.I. returned by the model.
K. Definitions of regressors.
L. Influence baseline (estimated from Control target stimulation in the no-task condition).
M,N. Influence shifts due to ongoing epoch/choice (estimated from Control target stimulation). The coefficients describing Early Cue and Late Cue are those reported in the main text panels Fig. 4J,L.
O. Influence shifts due to target class (estimated in the no-task condition). These results are similar to the results of no-task only influence analyses in Fig. S3I.
P,Q. Influence shifts due to the combination of target class (row) and epoch/choice (column), shown as a heat map. Non-significant coefficients (P_{CORR} > 0.05, Bonferroni 30X) are shown as white. The columns of coefficients corresponding to Early Cue and Late Cue are shown in Fig. 4K,M.

bioRxiv preprint doi: https://doi.org/10.64898/2026.04.07.716761; this version posted April 8, 2026. The copyright holder for this preprint (which was not certified by peer review) is the author/funder, who has granted bioRxiv a license to display the preprint in perpetuity. It is made available under aCC-BY 4.0 International license.

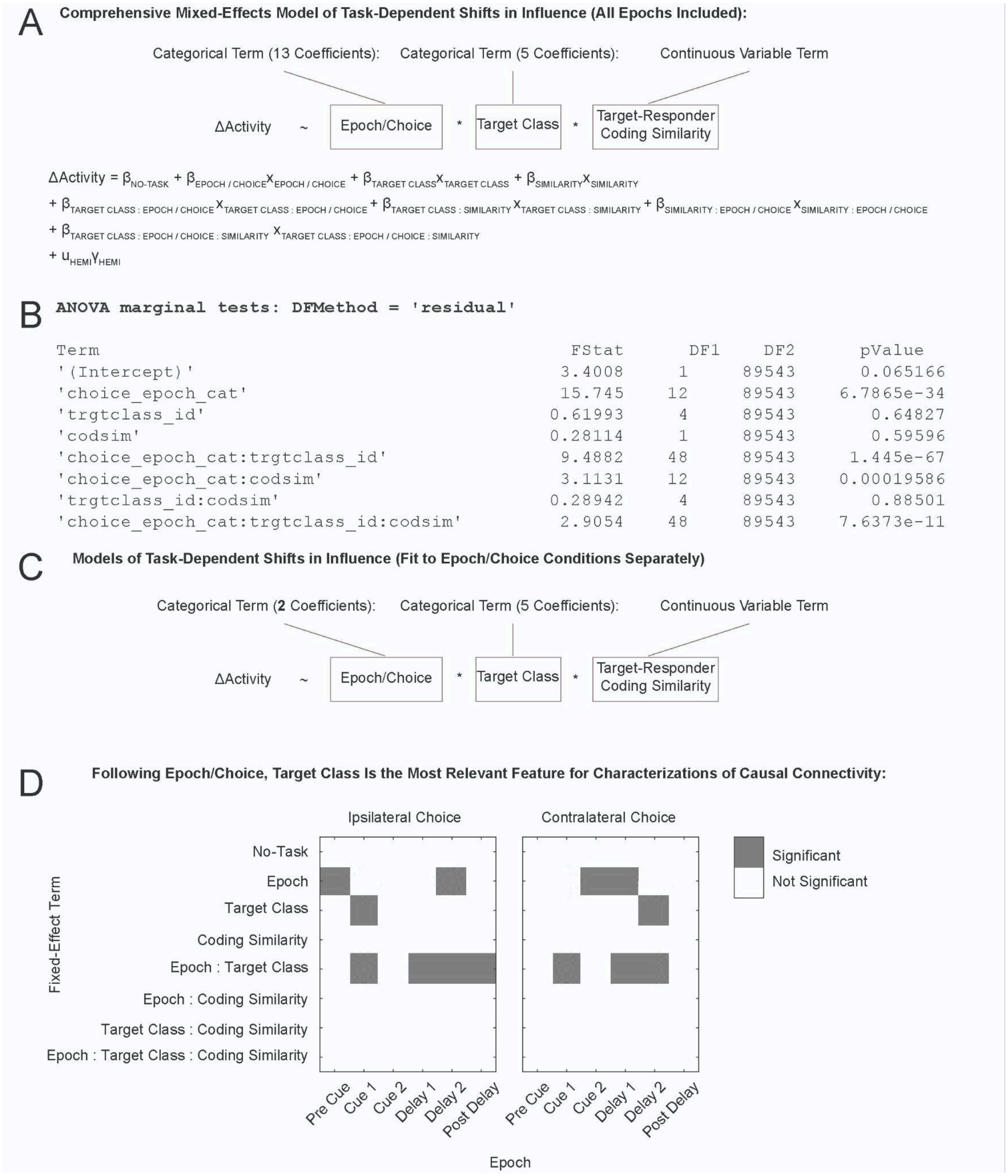

Term	FStat	DF1	DF2	pValue
'(Intercept)'	3.4008	1	89543	0.065166
'choice_epoch_cat'	15.745	12	89543	6.7865e-34
'trgtclass_id'	0.61993	4	89543	0.64827
'codsim'	0.28114	1	89543	0.59596
'choice_epoch_cat:trgtclass_id'	9.4882	48	89543	1.445e-67
'choice_epoch_cat:codsim'	3.1131	12	89543	0.00019586
'trgtclass_id:codsim'	0.28942	4	89543	0.88501
'choice_epoch_cat:trgtclass_id:codsim'	2.9054	48	89543	7.6373e-11

Figure S8. Feature Selection in Mixed-Effects Models of Influence, Related to Figure 4.

A. Description of the comprehensive mixed-effects model that incorporated all terms up to the joint three-way interaction term between epoch/choice, target class, and coding similarity (130 coefficients). Epoch/choice was parameterized with 13 coefficients (one no-task baseline, and twelve offsets for the 6 epochs * 2 choices). Target class was parameterized with 5 coefficients (one Control target stimulation

bioRxiv preprint doi: https://doi.org/10.64898/2026.04.07.716761; this version posted April 8, 2026. The copyright holder for this preprint (which was not certified by peer review) is the author/funder, who has granted bioRxiv a license to display the preprint in perpetuity. It is made available under aCC-BY 4.0 International license.

baseline and four target class specific offsets). Coding similarity, a continuous variable, was parameterized with 1 coefficient.

B. Results of the marginal tests (*F*-tests) on the fixed-effects terms (Matlab's **glme.anova**), which test whether the coefficients within each fixed-effect term significantly deviate from zero.

C. Description of the mixed-effects models estimated per epoch/choice condition. In contrast to panel A, two coefficients were necessary for epoch/choice, one to estimate the no-task baseline, and the other to estimate the epoch/choice-dependent shift.

D. The significance of terms which modulated the influence, calculated within epoch/choice conditions separately (panel C), evaluated with marginal tests (as in panel B). These were Bonferroni corrected (12 X), and reported in this panel if $P_{CORR} < 0.05$.

During the Early Cue, on ipsilateral trials, coding similarity was not significant (*F*-statistic = 0.01, *n.s.*) while the joint target class : epoch term was (*F*-statistic = 13.4, ****$P_{CORR} < 0.0001$). Similarly for contralateral trials (coding similarity *F*-statistic = 0.04, *n.s.*; target class : epoch *F*-statistic = 6.4, ***$P_{CORR} < 0.001$).

During the Late Cue causal connectivity was not modulated by target class (Epoch : Target Class term, ipsilateral choice trials, *F*-statistic = 2.3, *n.s.*; contralateral choice trials, *F*-statistic = 2.4, *n.s.*; Bonferroni 12X).

Thus ongoing epoch/choice and target class modulated influence most consistently, whereas coding similarity did not modulate influence significantly in any of the epochs.

bioRxiv preprint doi: https://doi.org/10.64898/2026.04.07.716761; this version posted April 8, 2026. The copyright holder for this preprint (which was not certified by peer review) is the author/funder, who has granted bioRxiv a license to display the preprint in perpetuity. It is made available under aCC-BY 4.0 International license.

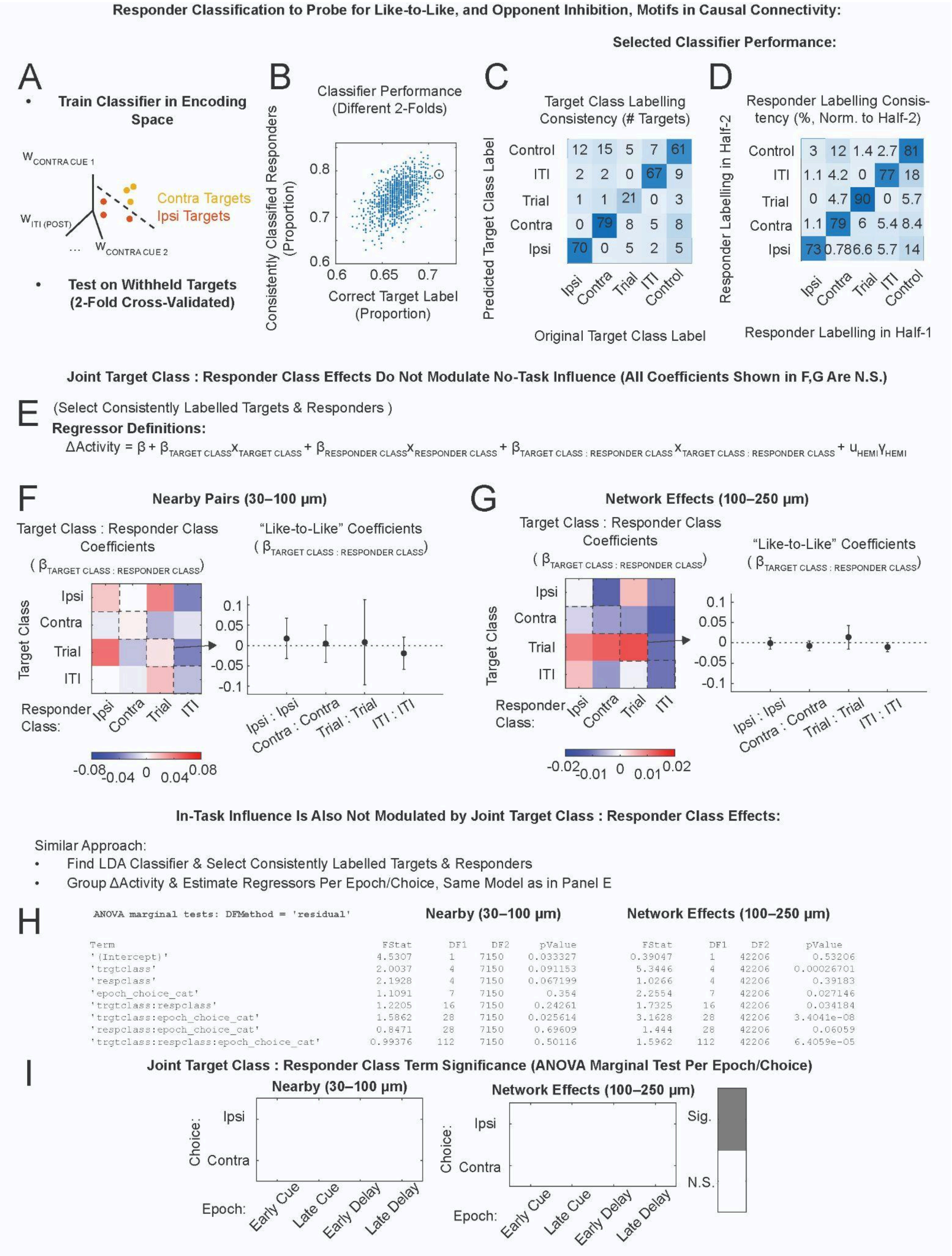

bioRxiv preprint doi: https://doi.org/10.64898/2026.04.07.716761; this version posted April 8, 2026. The copyright holder for this preprint (which was not certified by peer review) is the author/funder, who has granted bioRxiv a license to display the preprint in perpetuity. It is made available under aCC-BY 4.0 International license.

Figure S9. No Evidence of "Like-to-Like" or "Opponent Inhibition" Motifs in the Causal Connectivity, Related to Figures 4 and 5.

We classified responder neurons into the same encoding classes as the targets (Ipsi, Contra, Trial, ITI and Control), and then probed whether terms describing joint effects from target class and responder class modulated influence. Joint effect terms did not statistically deviate from zero. Additionally, the inferred coefficients were not consistent with like-to-like (positive terms between the same target and responder class) or opponent inhibition (negative terms between different classes, such as Ipsi targets : Contra responders) motifs. The approach and results are illustrated in greater detail for the no-task data (11 sessions, panels A–G), and then summarized for the in-task data (20 sessions, panels H–J).

A–D. Approach to responder categorization.

A. We estimated linear discriminant classifiers that separated the original target classes in the 21-dimensional regressor space of the single neuron GLMS (Fig. S2), using a 2-fold crossvalidated approach. In each fold, we tested labelling predictions on the withheld half of targets. We also used both folds to label responders and measure the consistency of responder labelling.

B. We repeated the data split and classification 1000X and selected the data split (indicated with black circle) that maximized the proportion of correctly labelled targets (x-axis). The two classifiers estimated from separate halves of this data split classified responders with ~80% similarity (y-axis).

C. Confusion matrix of target class labels in the selected classifier.

D. Similarity of responder labelling in the selected classifier, across halves, normalized by row. Total number of diagonal elements (from Ipsi to Control): 7080, 8566, 2417, 11122, 35284.

For subsequent analyses, we only included targets that were correctly classified, and responders that were consistently classified across the two halves of the data (i.e. diagonal elements in panels C and D; 46 228 pairs, or 45%, passed this criteria). These were then grouped by separation distance below.

E. Definitions of regressors.

F,G. Influence shifts due to the combination of target class and responder class. All of the coefficients shown in panels F and G were not significant; they are shown here to illustrate the lack of strong positive diagonal elements (like-to-like), as well as the lack of strong negative off-diagonal elements (opponent inhibition, e.g. between Ipsi and Contra).

F. Influence shifts estimated using target-responder pairs nearby (30–100 μm, ANOVA: F-statistic = 0.85, P = 0.6). This data from 8 hemispheres, 295 targets, 3410 pairs.

Left panel: All joint target class : responder class coefficients estimated by the model in panel E, shown as a heat map (row: target class; column: responder class).

Right panel: Coefficients describing the interaction between like-to-like elements (diagonal of the left panel). Error bars are 95% C.I.

G. Influence shifts estimated using target-responder pairs at network effects separations (100–250 μm, ANOVA: F-statistic = 1.4, P = 0.15). This data from 8 hemispheres, 298 targets, 19 496 pairs. Left and right panels as in panel F.

H–J. To similarly probe in-task influence for evidence of like-to-like motifs, we repeated the classification steps in panels A–D using the full 20-session dataset. This yielded a classification with [13072, 11666, 5748, 29518, 33608] consistently identified responders in the five categories as above; a total of 62 749 target-responder pairs (43%) passed correct-target and consistent-responder classification criteria. We then subselected the four best sampled epochs, Early Cue to Late Delay, to perform the analysis within epoch/choice conditions. This seemed the most appropriate approach given our primary finding in this paper, which is that ongoing computation is the most relevant feature in characterizations of causal connectivity.

We next searched for the 3-way joint effect between epoch, target class and responder class (panel H) and then subdivided the data by epoch/choice and searched for 2-way joint effects in target class and

bioRxiv preprint doi: https://doi.org/10.64898/2026.04.07.716761; this version posted April 8, 2026. The copyright holder for this preprint (which was not certified by peer review) is the author/funder, who has granted bioRxiv a license to display the preprint in perpetuity. It is made available under aCC-BY 4.0 International license.

responder class (panel **I**). The data was sampled from 11 hemispheres, 274 targets, 3 657 pairs (30–100 µm), and 11 hemispheres, 279 targets, 21 203 pairs (100–250 µm).
H. Statistics of the terms in the comprehensive mixed-effects model (ANOVA marginal tests), for nearby (left columns) and network effects (right columns) pairs. The joint 3-way term between target class, responder class, and epoch/choice was found to be statistically significant. However, individual coefficients (not shown) were not significant following multiple comparisons correction (30X). Also it is noteworthy that of the three 2-way interaction terms, target class : epoch was highly significant, while the other two terms were borderline, or not at all, significant. This suggests caution in interpreting the significance of the 3-way term naively, and that the significance of the joint 3-way term may be inherited from the significance of the 2-way target class : epoch term. For this reason we subsequently performed similar analyses within separate epoch/choice conditions (panel **I**).
I. Significance of the joint target class : responder class term (ANOVA marginal tests), evaluated per epoch/choice condition separately. The joint term did not significantly deviate from zero in any of the conditions (all $P_{\mathrm{CORR}} > 0.05$, Bonferroni, 12X). This was true for both nearby (left panel) and network effects (right panel) pairs.

bioRxiv preprint doi: https://doi.org/10.64898/2026.04.07.716761; this version posted April 8, 2026. The copyright holder for this preprint (which was not certified by peer review) is the author/funder, who has granted bioRxiv a license to display the preprint in perpetuity. It is made available under aCC-BY 4.0 International license.

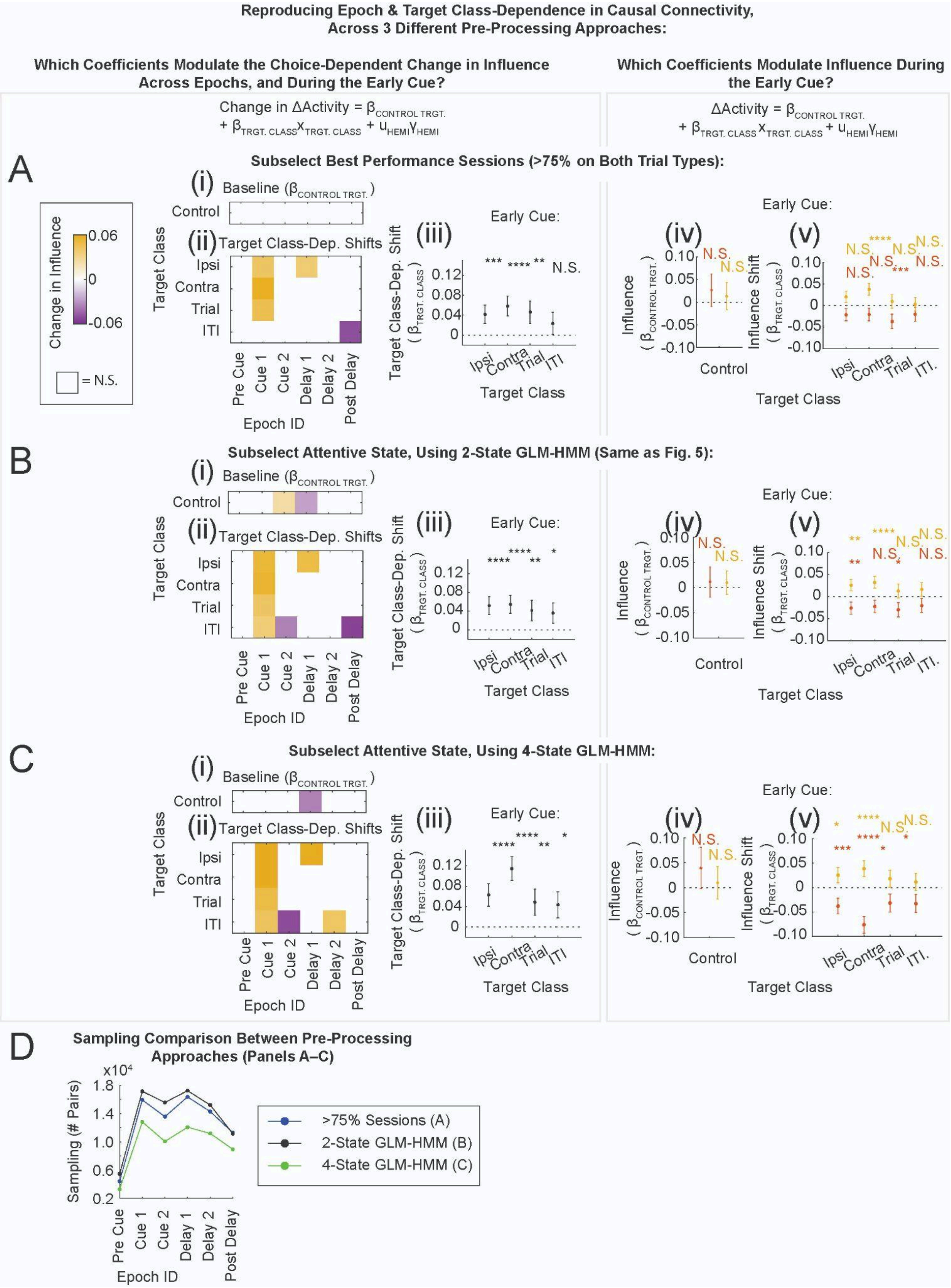

bioRxiv preprint doi: https://doi.org/10.64898/2026.04.07.716761; this version posted April 8, 2026. The copyright holder for this preprint (which was not certified by peer review) is the author/funder, who has granted bioRxiv a license to display the preprint in perpetuity. It is made available under aCC-BY 4.0 International license.

Figure S10. Target Class & Epoch-Dependent Shifts in Influence Are Consistent Across Attentive State Definitions, Related to Figure 5.

To get a sense for how strongly our results depended on choices of preprocessing, we repeated the analyses of epoch & target class dependencies in influence following two other preprocessing approaches. For a particular set of selection criteria (panels A–C, described below), we evaluated the dependence of the choice-dependent change in influence on target class, per epoch (left column, compare to Fig. 5D–F). We also reevaluated the dependence of influence on target class, per choice condition during the Early Cue (right column, compare to Fig. S11B). All significance indicators were corrected for multiple comparisons as in the main text.

A. We selected high performance sessions (mice ran >75% on both right- and left-trial types), and kept all non-aberrant post warm-up trials as described in Methods: Behavioral Trial Selection.

B. We used the two-state GLM-HMM (Fig. S4) to select the attentive state (reproduced from Fig. 5 and the main text).

C. We used a four-state GLM-HMM to select a higher-performing attentive state than in the two-state GLM-HMM model.

In all three approaches (panels A–C), Control target stimulation did not significantly modulate the choice-dependent change in influence during the Early Cue (i, left column). Target class modulated the choice-dependent change in influence primarily during the Early Cue, and not in other epochs (ii, left column). The effects from Ipsi, Contra and Trial target stimulation were most consistent, while the effect from ITI target stimulation was borderline (iii, left column).

Examining the influence by choice condition separately, Control target stimulation was not significant on ipsilateral or contralateral choice trials (iv, right column). Target class-dependent shifts in the influence were positive on contralateral trials (v, right column, gold) and negative on ipsilateral trials (v, right column, red).

D. Sampling across epochs for the three different pre-processing approaches shown in panels A–C.

bioRxiv preprint doi: https://doi.org/10.64898/2026.04.07.716761; this version posted April 8, 2026. The copyright holder for this preprint (which was not certified by peer review) is the author/funder, who has granted bioRxiv a license to display the preprint in perpetuity. It is made available under aCC-BY 4.0 International license.

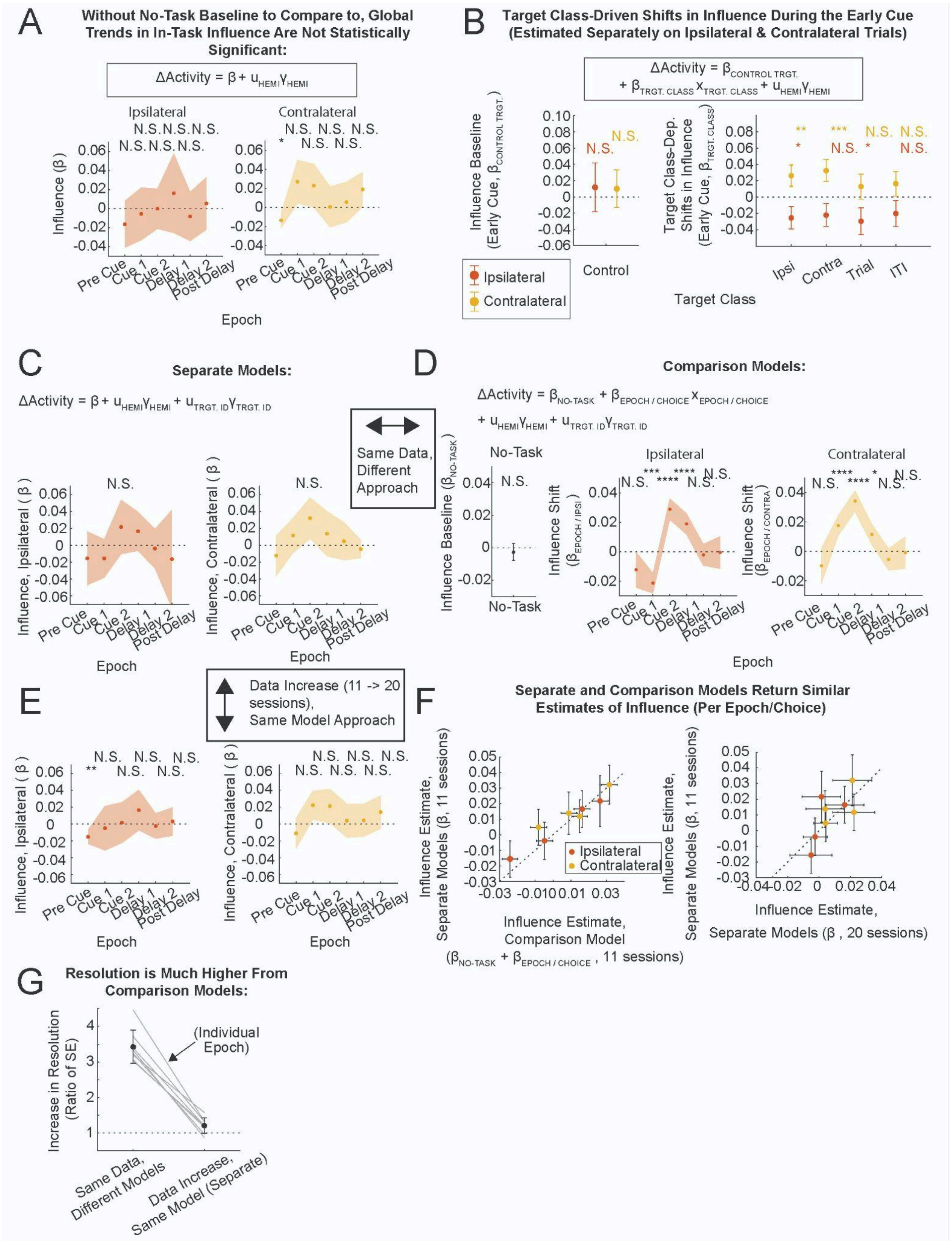

Figure S11. Supplemental Information Related To Figure 5.

bioRxiv preprint doi: https://doi.org/10.64898/2026.04.07.716761; this version posted April 8, 2026. The copyright holder for this preprint (which was not certified by peer review) is the author/funder, who has granted bioRxiv a license to display the preprint in perpetuity. It is made available under aCC-BY 4.0 International license.

A. Influence per epoch/choice, calculated with separate models per condition (Model 4.1, Table 1: asterisks indicate 12X Bonferroni corrected significance values).
B. Target class-dependent shifts in the influence, relative to Control target stimulation, during the Early Cue (Model 4.2, Table 1: asterisks indicate 60X Bonferroni corrected significance values). Note that some of these target class-dependent shifts were significant even though averaged trends in influence were not significant without the no-task comparison (panel A). These shifts were estimated separately within ipsilateral (red) and contralateral (gold) choice conditions. Differences between conditions (e.g. within Ipsi targets in the right panel) were similar to the absolute choice-dependent changes in influence reported in Fig. 5F; the number of random-effects coefficients was doubled in this analysis.
C–G. Models that estimated shifts in influence (e.g. Fig. 3I–K, Fig. 4C–E) had a much lower uncertainty on the estimate of the shift, than the uncertainty that we obtained in estimating absolute values (e.g. panel A here, or Fig. S5C–E). The drawback is that comparison models can not probe whether the absolute value of influence is net-excitatory or net-inhibitory. Here we quantified the reduction in uncertainty across different approaches, to clear up any confusion about the data.
C. In-task influence vs. epoch, estimated per epoch/choice (same data as Model 3.0, Table 1).
D. Influence baseline (no-task, left panel) and task-dependent influence shifts (ipsilateral choice, middle panel; contralateral choice, right panel), reproduced from Fig. 4C–E. These terms were estimated using the same data as in panel C. Note the similar epoch-dependent trends, and the significant reduction in uncertainty, compared to panel C.
E. In-task influence vs. epoch, estimated identically to panel C, using all the in-task data (same data as panel A; Model 4.1, Table 1; note the additional random-effects term compared to panel A).
F. Estimates of the in-task influence, comparing the different approaches in panels C–E. Each point is one of the four Cue / Delay epochs. Dashed line is unity: the different approaches yielded similar estimates.
Left panel: influence estimates from panels C (y-axis) and D (x-axis).
Right panel: influence estimates from panels C (y-axis) and E (x-axis).
G. Comparison of reduction in uncertainty between switching models with the same data (left side of panel; comparing panels D to C; median, 3.3x; range, 3.0–4.5x) and keeping the same model while increasing data sampling (right side of panel; comparing panels E to C; median, 1.2x; range, 0.9–1.6x; data increase from 8 to 11 hemispheres, 300 to 590 targets, 43 883 to 81 734 pairs). Each point is a choice-dependent Cue or Delay epoch, black is average, error bars are standard deviation over epochs.

bioRxiv preprint doi: https://doi.org/10.64898/2026.04.07.716761; this version posted April 8, 2026. The copyright holder for this preprint (which was not certified by peer review) is the author/funder, who has granted bioRxiv a license to display the preprint in perpetuity. It is made available under aCC-BY 4.0 International license.

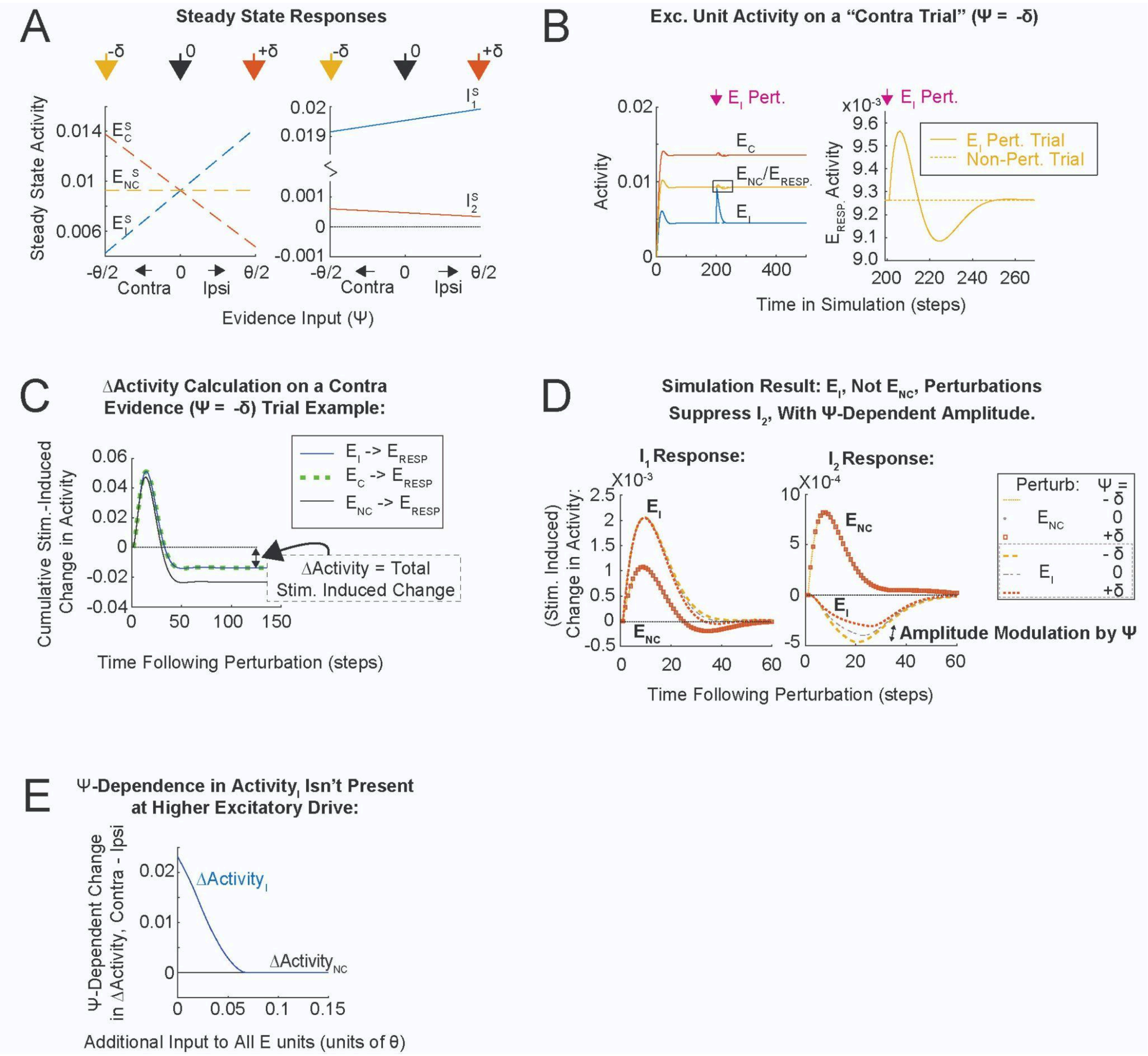

Figure S12. Simulation of the Conceptual Model, Related To Figure 6.

A. Steady state responses of excitatory (left panel) and inhibitory (right panel) units, in response to varying the evidence input (*Ψ*). In this model, the low activity levels in I_2 were key for *Ψ*-dependence in ΔActivity.

B. Excitatory unit activity during contra evidence (*Ψ*<0) trials. Full simulation (left panel), and zoom in on the response of E_{RESP} following stimulation (right panel) on an E_I-perturbation trial (solid line) and non-perturbation trial (dashed line). The difference between these two is the stimulation-induced change in Activity. The time-dependent decay following a response to perturbation reflects the underdamped regime: pathways involving higher numbers of synapses contribute progressively less to ΔActivity.

C. Cumulative stimulation-induced change in the activity of E_{RESP}. In the simulation, ΔActivity is defined as the total difference between stimulation and non-stimulation trials (summed over time), which can be visualized on this plot as the value of the cumulative as t -> infinity. By construction, ΔActivity following E_I (solid blue) and E_C (dashed green) perturbations is the same and different to ΔActivity following E_{NC} perturbations (solid black).

bioRxiv preprint doi: https://doi.org/10.64898/2026.04.07.716761; this version posted April 8, 2026. The copyright holder for this preprint (which was not certified by peer review) is the author/funder, who has granted bioRxiv a license to display the preprint in perpetuity. It is made available under aCC-BY 4.0 International license.

D. Stimulation-induced change in the activity of I_1 and I_2, in response to the two different perturbations in the three different Ψ conditions.
Left panel: I_1 was excited by both types of perturbations (E_I and E_{NC}).
Right panel: I_2 was excited by E_{NC} perturbations and suppressed by E_I perturbations. As suggested by the schematic in Fig. 6F, the magnitude of the I_2 response to E_I perturbations was Ψ-dependent.
E. Ψ-dependent change in ΔActivity, evaluated at Ψ = +/- δ, vs. additional input to all excitatory units. Additional input of 0 is the default setting (in panels A–D). Providing additional input to all excitatory units moved I_2 away from threshold, and removed the Ψ-dependence in $\Delta Activity_I$ (blue line). $\Delta Activity_{NC}$ (black line) shown for comparison.

bioRxiv preprint doi: https://doi.org/10.64898/2026.04.07.716761; this version posted April 8, 2026. The copyright holder for this preprint (which was not certified by peer review) is the author/funder, who has granted bioRxiv a license to display the preprint in perpetuity. It is made available under aCC-BY 4.0 International license.

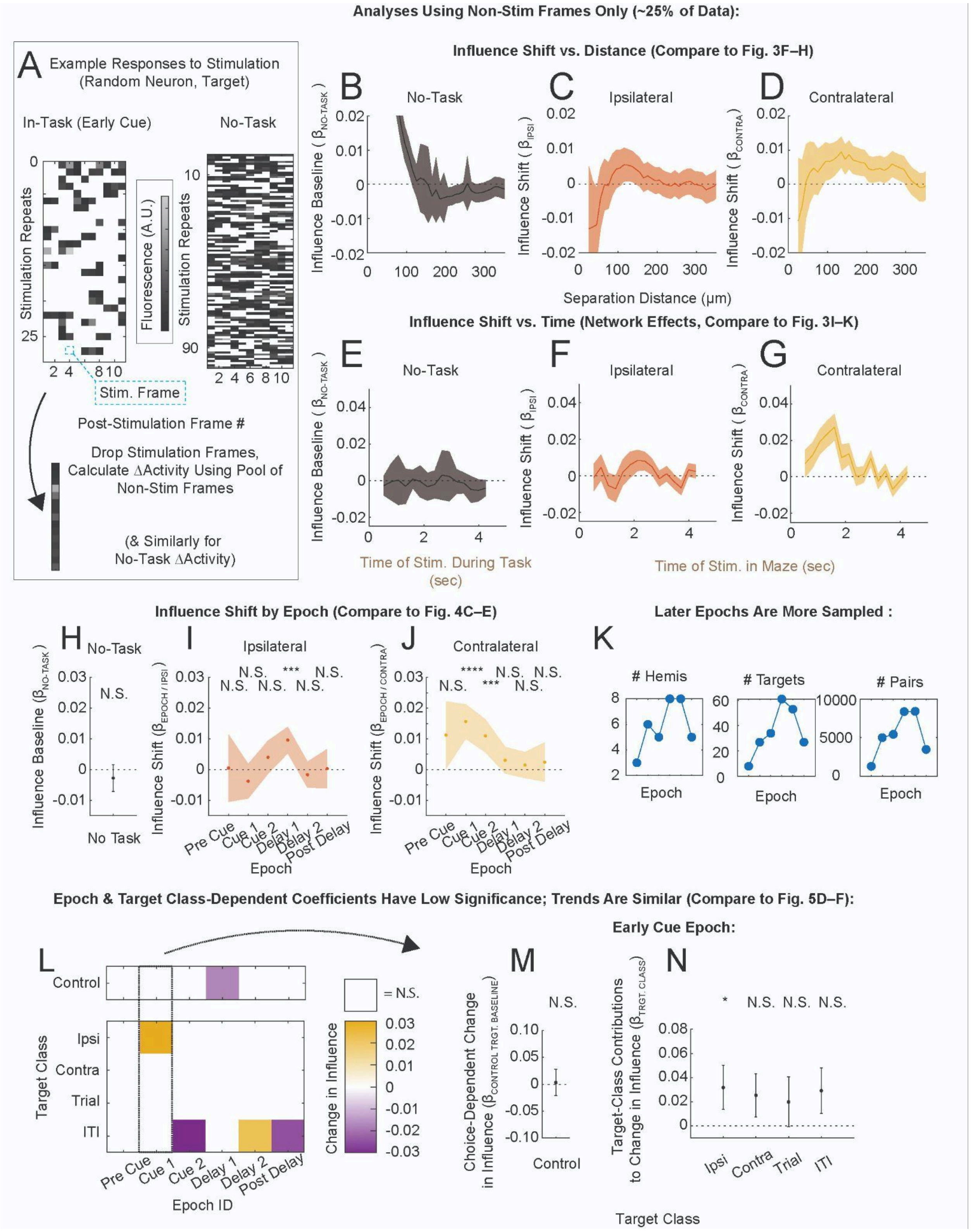

Figure S13. Properties of Causal Connectivity Estimated Using Only Non-Stimulation Imaging Frames Are Consistent With the Rest of the Paper, Related to Figures 3–5.

bioRxiv preprint doi: https://doi.org/10.64898/2026.04.07.716761; this version posted April 8, 2026. The copyright holder for this preprint (which was not certified by peer review) is the author/funder, who has granted bioRxiv a license to display the preprint in perpetuity. It is made available under aCC-BY 4.0 International license.

In these analyses we explicitly removed imaging frames with simultaneous stimulation (~75% of the recorded data in some cases), and recalculated the main features of influence presented in Fig. 3–5 (Methods : Stimulation-Induced Artefacts During Imaging).
A. Fluorescence of a responder neuron in response to stimulation of a particular target (in-task example, left panel; no-task, right panel). Here, frames with simultaneous stimulation were set to white. We subselected imaging frames without simultaneous stimulation in the 1–3 seconds following stimulation (the same time limits as in other analyses in this paper). These were concatenated into a pool of post-stim frames. In this approach the stimulation and comparison responses, as well as the normalization coefficient, were calculated with respect to one frame. In all other analyses in this paper we calculated these quantities within the 2-second post-stimulation average (11 frames). These changes scaled the magnitude of the normalization coefficient, and subsequently the influence. Additionally, we replaced the sufficient sampling criterion based on trials (at least 10 stim and 10 comparison trials) with a criterion on the number of non-stimulation frames (at least 40 following stim and 40 in the comparison condition). Otherwise, the analyses here were identical to those in the rest of the paper. Because the stimulation rate on the timescale of a trial was different between in-task and no-task experiments (there was no stimulation during the ITI in-task, Methods : In-Task Stimulation), there were more non-stimulation imaging frames following stimulation in the no-task experiment. In-task, the response to stimulation during the early epochs overlapped with stimulation on 75% of trials (see also panel K).
B–D. Influence baseline (**B**) and influence shifts (**C,D**) vs. distance, compare to Fig. 3F–H.
E–G. (Network Effects) Influence baseline (**E**) and influence shifts (**F,G**) vs. time of stimulation, compare to Fig. 3I–K.
H–J. (Network Effects). Influence baseline (**H**) and influence shifts (**I,J**) vs. epoch, compare to Fig. 4C–E.
K. Sampling of hemispheres, targets and pairs used in panels H–J. In these analyses, the Pre Cue, Early and Late Cue epochs had ~25% of the data available to estimate ΔActivity. Because ΔActivity is evaluated in the 1–3 second bin following stimulation, later epochs have an increased proportion of non-stimulation imaging frames. Estimates of ΔActivity in the Post-Delay were calculated using imaging frames following completion of the stimulation sequences and were essentially unaffected by the subselection in this analysis.
L–M. Epoch & target class-dependent analysis of the choice-dependent change in influence, compare to Fig. 5D–F.
L. Shifts in the choice-dependent change in influence due to target class-specific stimulation, estimated per epoch, shown as a heat map (row: target class; column: epoch). Coefficients which were not significant (P_{CORR}>0.05) are shown in white.
M. Baseline choice-dependent change in influence, during the Early Cue, measured by Control target class stimulation (compare to Early Cue in Fig. 5D)
N. Target class-dependent contributions to the choice-dependent shift in influence, during the Early Cue (compare to Fig. 5F).
As in Fig. 5, error bars in panels M,N are the uncorrected 95% C.I.; asterisks indicate multiple comparisons corrected significance values (Bonferroni, 30X).
To summarize, these results were consistent with the analyses that included imaging frames with stimulation and which were reported throughout this paper. On their own, these results become less conclusive as the data is increasingly subdivided (especially by both epoch and target class, panels L–N), because of the greatly reduced sampling.

bioRxiv preprint doi: https://doi.org/10.64898/2026.04.07.716761; this version posted April 8, 2026. The copyright holder for this preprint (which was not certified by peer review) is the author/funder, who has granted bioRxiv a license to display the preprint in perpetuity. It is made available under aCC-BY 4.0 International license.

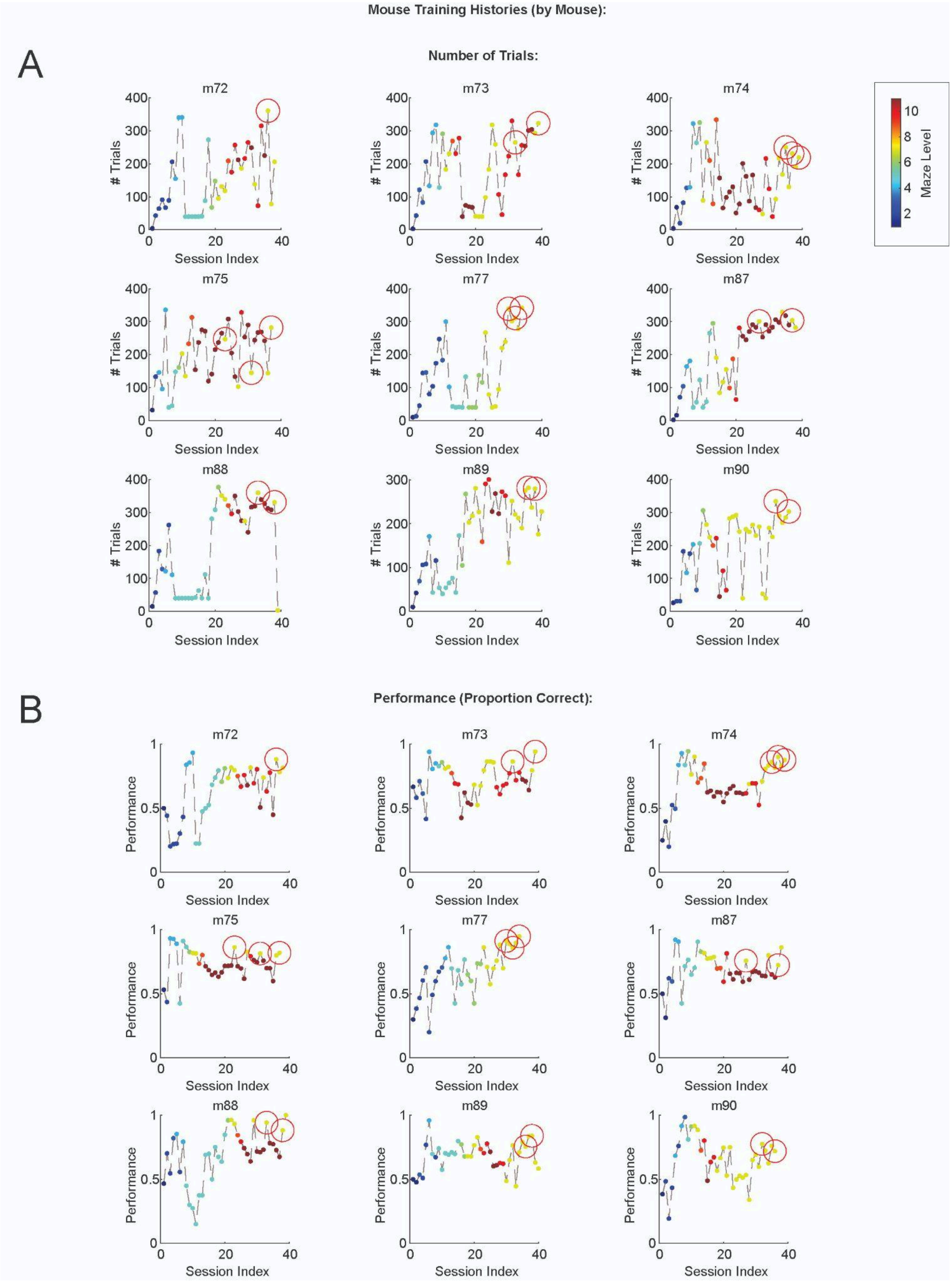

bioRxiv preprint doi: https://doi.org/10.64898/2026.04.07.716761; this version posted April 8, 2026. The copyright holder for this preprint (which was not certified by peer review) is the author/funder, who has granted bioRxiv a license to display the preprint in perpetuity. It is made available under aCC-BY 4.0 International license.

Figure S14. Training Histories of the Mice Involved in Single-Cell Perturbation Experiments, Related to Figure 1.

Single-cell stimulation sessions are indicated with red circles. Mice were trained up to T11 (full difficulty ATT maze with distractors), though not all mice reached T11. All animals were trained primarily for a series of multi-neuron perturbation experiments, that have not yet been fully analyzed and which we plan to publish separately (not indicated on these panels). Single-cell stimulation experiments occurred after the multi-neuron perturbation experiments, at the end of the experimental round with the mice. These were performed in T7 (no-distractors version of the ATT maze).

A. Number of trials run per session, maze level indicated by color.

B. Performance (% Correct, over all trials, including warm up and aberrant trials) per session, maze level indicated by color.

REFERENCES

1. Chen, T.-W. *et al.* Ultrasensitive fluorescent proteins for imaging neuronal activity. *Nature* **499**, 295–300 (2013).
2. Klapoetke, N. C. *et al.* Independent optical excitation of distinct neural populations. *Nat. Methods* **11**, 338–346 (2014).
3. Baker, C. A., Elyada, Y. M., Parra, A. & Bolton, M. M. Cellular resolution circuit mapping with temporal-focused excitation of soma-targeted channelrhodopsin. *eLife* **5**, e14193 (2016).
4. Marshel, J. H. *et al.* Cortical layer–specific critical dynamics triggering perception. *Science* **365**, eaaw5202 (2019).
5. LaFosse, P. K. *et al.* Bicistronic Expression of a High-Performance Calcium Indicator and Opsin for All-Optical Stimulation and Imaging at Cellular Resolution. *eNeuro* **10**, (2023).
6. Pachitariu, M. *et al.* Suite2p: beyond 10,000 neurons with standard two-photon microscopy. 061507 Preprint at https://doi.org/10.1101/061507 (2017).
7. Botcherby, E. J., Juškaitis, R., Booth, M. J. & Wilson, T. An optical technique for remote focusing in microscopy. *Opt. Commun.* **281**, 880–887 (2008).
8. Friedrich, J., Zhou, P. & Paninski, L. Fast online deconvolution of calcium imaging data. *PLOS Comput. Biol.* **13**, e1005423 (2017).
9. Bolkan, S. S. *et al.* Opponent control of behavior by dorsomedial striatal pathways depends on task demands and internal state. *Nat. Neurosci.* **25**, 345–357 (2022).

bioRxiv preprint doi: https://doi.org/10.64898/2026.04.07.716761; this version posted April 8, 2026. The copyright holder for this preprint (which was not certified by peer review) is the author/funder, who has granted bioRxiv a license to display the preprint in perpetuity. It is made available under aCC-BY 4.0 International license.

Table S1.

Mixed-effects models are indicated with model name (formulas, sample sizes and other details can be found in Table 1). All statistical comparisons in mixed-effects models are against a zero baseline. ANOVA marginal tests tested the significance of fixed-effects terms (across multiple coefficients), against a zero baseline. *P*-values reported in this table were not adjusted for multiple comparisons; correction factors are given here for the corrected significance values reported in the main text (indicated as P_{CORR}).

Row	Panel	Details	Model Name	Estimate (β)	95% CI	*t* stat	*d f*	*P*, uncorrected	Bonferroni Correction Factor
1.	2i	in-task influence, direct activations (0–20um separation)	1.0	0.48	[0.39 0.57]	10.2	751	9e-23	
2.	3g	in-task influence shift, 150um, ipsilateral trials	1.0	0.010	[0.004 0.015]	3.6	‘’	4e-04	
3.	3h	(‘’), contralateral trials	1.0	0.013	[0.007 0.018]	4.6	‘’	4e-06	
4.	3i	no-task influence baseline, network effects, at ~1 sec (stim. bins 3–6)	1.2	-0.002	[-0.010 0.006]	-0.4	33897	0.69	
5.	3j	ipsi influence shifts, network effects, at ~1 sec (stim. bins 3–6)	1.2	-0.013	[-0.020 -0.007]	-4.0	33897	6e-05	9X
6.	3j	(‘’), at ~ 1.8 sec (stim. bins 6–9)	1.2	0.026	[0.019 0.032]	7.4	33597	1e-13	9X
7.	3k	contra influence shifts, network effects, stim. bins 3–6	1.2	0.031	[0.024 0.037]	9.2	33897	3e-20	9X
8.	3k	(‘’), stim. bins 6–9	1.2	0.037	[0.030 0.044]	10.8	33597	5e-27	9X
9.	s5e	contra influence, network effects, stim. bins 6–9	1.3	0.042	[0.024 0.060]	4.5	11199	6.8e-06	9X
10.	3m	ipsi excitability shifts, direct activations (0–25um sep.), stim. bins 3–6	1.2	+0.01	[-0.11 +0.13]	0.2	255	0.86	
11.		two sample t-test; ipsi excitability comparison, direct activations (0–25um sep.), stim bins 3–6 vs. stim bins 6–9				0.5	149	0.64	
12.		two sample t-test; contra excitability comparison, direct activations (0–25um sep.), stim bins 3–6 vs. stim bins 12–15				-0.2	156	0.87	
13.	s5m	ipsi binarized influence shift, network effects, stim. bins 3–6.	1.4	0.00	[-0.029 0.023]	-0.2	33897	0.81	9X
14.	s5m	(‘’), stim. bins 6–9.	1.4	0.09	[0.06 0.11]	6.7	33597	2e-11	9X
15.	s5n	contra binarized influence shift, network effects, stim. bins 3–6.	1.4	0.12	[0.10 0.15]	9.1	33897	7e-20	9X
16.	s5n	(‘’), stim. bins 6–9.	1.4	0.11	[0.08 0.13]	8.1	33597	5e-16	9X
17.	s5q	contra binarized influence, network effects, at ~1.8 sec (stim. bins 6–9).	1.5	0.11	[0.06 0.16]	4.0	11199	6e-05	9X

bioRxiv preprint doi: https://doi.org/10.64898/2026.04.07.716761; this version posted April 8, 2026. The copyright holder for this preprint (which was not certified by peer review) is the author/funder, who has granted bioRxiv a license to display the preprint in perpetuity. It is made available under aCC-BY 4.0 International license.

18.	s6d	ipsi comparison,<br>influence,<br>evidence threshold = 1.5,<br>ambiguous	2.0	-0.013	[-0.012<br>0.09]	-0.24	788	0.81	
19.	s6e	(‘’)<br>more evidence	2.0	-0.020	[-0.13<br>0.09]	-0.36	‘’	0.72	
20.	s6g	contra comparison,<br>influence,<br>evidence threshold = 1.5,<br>ambiguous	2.0	-0.10	[-0.19<br>-0.02]	-2.4	696	0.014	
21.	s6h	(‘’)<br>more evidence	2.0	+0.13	[0.036<br>0.22]	2.7	‘’	0.007	
22.	s6j	ipsi comparison,<br>evidence threshold = 3.5,<br>baseline influence (β_{AMB})	2.1	-0.08	[-0.12<br>-0.039]	-3.8	1970	2e-04	
23.	s6k	(‘’)<br>influence shift (β_{EV})	2.1	+0.11	[0.06<br>0.16]	4.3	‘’	2e-05	
24.	s6l	contra comparison,<br>evidence threshold = 1.5,<br>baseline influence (β_{AMB})	2.1	-0.13	[-0.17<br>-0.09]	-6.0	1392	2e-09	
25.	s6m	(‘’)<br>influence shift (β_{EV})	2.1	+0.29	[0.23<br>0.35]	10.0	‘’	8e-23	
26.	4d	ipsi influence shift, Cue 1	3.0	-0.021	[-0.028<br>-0.015]	-6.2	1.3e+<br>05	8e-10	12X
27.	4d	ipsi influence shift, Cue 2	3.0	0.029	[0.022<br>0.036]	7.9	‘’	2e-15	12X
28.	4d	ipsi influence shift, Delay 1	3.0	0.019	[0.012<br>0.026]	5.2	‘’	2e-07	12X
29.	4e	contra influence shift, Cue 1	3.0	0.018	[0.011<br>0.024]	5.0	‘’	5e-07	12X
30.	4e	contra influence shift, Cue 2	3.0	0.034	[0.027<br>0.041]	9.4	‘’	8e-21	12X
31.	4e	contra influence shift, Delay 1	3.0	0.011	[0.004<br>0.019]	3.2	‘’	1.5e-03	12X
32.		two-sample t-test;<br>ipsi excitability comparison,<br>direct activations (0–20um sep.),<br>Cue 1 vs. Cue 2				1.0	88	0.30	
33.		two-sample t-test;<br>contra excitability comparison,<br>direct activations (0–20um sep.),<br>Cue 1 vs. Delay 2				1.1	84	0.29	
34.	s7e	two-sample t-test;<br>speed comparison: ipsilateral vs. contralateral choice trials.<br>Tests were done per-epoch, using per-session averages. Values are given for the 6 epochs (Pre Cue to Post Delay), in order.				-0.07,<br>-0.17,<br>-0.06,<br>0.56,<br>1.60,<br>2.48	38,38<br>,38,3<br>8,38,<br>36	0.95,<br>0.86,<br>0.95,<br>0.58,<br>0.12,<br>0.02	
35.	s8d	ANOVA marginal test on term: coding similarity,<br>in Cue 1, went-ipsi. *df*1 = 1.				*F*-stat<br>0.01	1501<br>0	0.9	12X
36.	s8d	ANOVA marginal test on term: target class:epoch,<br>in Cue 1, went-ipsi. *df*1 = 1.				*F*-stat<br>13.4	‘’	7e-11	12X
37.	s8d	ANOVA marginal test on term: coding similarity,<br>in Cue 1, went-contra. *df*1 = 1.				*F*-stat<br>0.04	‘’	0.84	12X
38.	s8d	ANOVA marginal test on term: target class,<br>in Cue 1, went-contra. *df*1 = 1.				*F*-stat<br>6.4	‘’	4e-05	12X
39.	s8d	ANOVA marginal test on term: target class:epoch,<br>in Cue 2, went-ipsi. *df*1 = 1.				*F*-stat<br>2.3	1232<br>4	0.059	12X
40.	s8d	ANOVA marginal test on term: target class:epoch,<br>in Cue 2, went-contra. *df*1 = 1.				*F*-stat<br>2.4	‘’	0.047	12X

bioRxiv preprint doi: https://doi.org/10.64898/2026.04.07.716761; this version posted April 8, 2026. The copyright holder for this preprint (which was not certified by peer review) is the author/funder, who has granted bioRxiv a license to display the preprint in perpetuity. It is made available under aCC-BY 4.0 International license.

41.	4j	$\beta_{\text{CUE1 / IPSILATERAL CHOICE}}$	3.1	0.001	[-0.010 0.012]	0.2	1.3e05	0.82	30X
42.	4j	$\beta_{\text{CUE1 / CONTRALATERAL CHOICE}}$	‘’	-0.007	[-0.018 0.004]	-1.2	‘’	0.24	30X
43.	4k	$\beta_{\text{IPSI TRGT : CUE1 / IPSILATERAL CHOICE}}$	‘’	-0.012	[-0.028 0.005]	-1.4	‘’	0.15	30X
44.	4k	$\beta_{\text{CONTRA TRGT : CUE1 / IPSILATERAL CHOICE}}$	‘’	-0.055	[-0.072 -0.039]	-6.5	‘’	8e-11	30X
45.	4k	$\beta_{\text{TRIAL TRGT : CUE1 / IPSILATERAL CHOICE}}$	‘’	-0.040	[-0.061 -0.019]	-3.8	‘’	1e-04	30X
46.	4k	$\beta_{\text{ITI TRGT : CUE1 / IPSILATERAL CHOICE}}$	‘’	-0.030	[-0.047 -0.013]	-3.4	‘’	6e-04	30X
47.	4k	$\beta_{\text{IPSI TRGT : CUE1 / CONTRALATERAL CHOICE}}$	‘’	0.034	[0.018 0.050]	4.1	‘’	4e-05	30X
48.	4k	$\beta_{\text{CONTRA TRGT : CUE1 / CONTRALATERAL CHOICE}}$	‘’	0.030	[0.013 0.047]	3.5	‘’	4e-04	30X
49.	4k	$\beta_{\text{TRIAL TRGT : CUE1 / CONTRALATERAL CHOICE}}$	‘’	0.040	[0.019 0.060]	3.8	‘’	2e-04	30X
50.	4k	$\beta_{\text{ITI TRGT : CUE1 / CONTRALATERAL CHOICE}}$	‘’	0.019	[0.002 0.036]	2.2	‘’	0.03	30X
51.	4l	$\beta_{\text{CUE2 / IPSILATERAL CHOICE}}$	‘’	0.021	[0.010 0.032]	3.8	‘’	2e-4	30X
52.	4l	$\beta_{\text{CUE2 / CONTRALATERAL CHOICE}}$	‘’	0.049	[0.038 0.058]	8.8	‘’	9e-19	30X
53.	4m	$\beta_{\text{IPSI TRGT : CUE2 / IPSILATERAL CHOICE}}$	‘’	0.014	[-0.002 0.030]	1.7	‘’	0.09	30X
54.	4m	$\beta_{\text{CONTRA TRGT : CUE2 / IPSILATERAL CHOICE}}$	‘’	0.006	[-0.010 0.023]	0.7	‘’	0.45	30X
55.	4m	$\beta_{\text{TRIAL TRGT : CUE2 / IPSILATERAL CHOICE}}$	‘’	0.044	[0.021 0.067]	3.8	‘’	0.0001	30X
56.	4m	$\beta_{\text{ITI TRGT : CUE2 / IPSILATERAL CHOICE}}$	‘’	0.015	[-0.003 0.033]	1.6	‘’	0.11	30X
57.		ANOVA marginal test on term: coding similarity (relevant features for choice-dependent change in influence, during the Early Cue) *df1* = 1.	4.3			*F*-stat 0.0	12519	0.96	
58.		ANOVA marginal test on term: target class (relevant features for choice-dependent change in influence, during the Early Cue) *df*1 = 4.	4.3			*F*-stat 14.0	12519	2e-11	
59.	5d	baseline choice-dependent change in influence, Early Cue	4.0	-0.002	[-0.031 0.027]	-0.1	17129	0.90	30X
60.	5e,f	target class-specific shifts in the choice-dependent change in influence, Early Cue, β_{IPSI}	‘’	0.052	[0.033 0.071]	5.3	‘’	9e-08	‘’
61.	5e,f	‘’ , β_{CONTRA}	‘’	0.054	[0.035 0.074]	5.5	‘’	3e-08	‘’
62.	5e,f	‘’ , β_{TRIAL}	‘’	0.041	[0.019 0.064]	3.6	‘’	0.0003	‘’
63.	5e,f	‘’ , β_{ITI}	‘’	0.036	[0.014 0.058]	3.3	‘’	0.001	‘’
